# Induced Pluripotent Stem Cell for the Study and Treatment of Sickle Cell Anemia

**DOI:** 10.1155/2017/7492914

**Published:** 2017-07-26

**Authors:** Luiza Cunha Junqueira Reis, Virgínia Picanço-Castro, Bárbara Cristina Martins Fernandes Paes, Olívia Ambrozini Pereira, Isabela Gerdes Gyuricza, Fabiano Tófoli de Araújo, Mariana Morato-Marques, Lílian Figueiredo Moreira, Everton de Brito Oliveira Costa, Tálita Pollyanna Moreira dos Santos, Dimas Tadeu Covas, Lygia da Veiga Pereira Carramaschi, Elisa Maria de Sousa Russo

**Affiliations:** ^1^Pharmaceutical Sciences School of Ribeirão Preto, University of São Paulo, Ribeirão Preto, SP, Brazil; ^2^Blood Center Foundation of Ribeirão Preto, University of São Paulo, Ribeirão Preto, SP, Brazil; ^3^Medical School of Ribeirão Preto, University of São Paulo, Ribeirão Preto, SP, Brazil; ^4^Philosophy, Sciences and Languages School of Ribeirão Preto, University of São Paulo, Ribeirão Preto, SP, Brazil; ^5^Institute of Biosciences, University of São Paulo, São Paulo, SP, Brazil

## Abstract

Sickle cell anemia (SCA) is a monogenic disease of high mortality, affecting millions of people worldwide. There is no broad, effective, and safe definitive treatment for SCA, so the palliative treatments are the most used. The establishment of an in vitro model allows better understanding of how the disease occurs, besides allowing the development of more effective tests and treatments. In this context, iPSC technology is a powerful tool for basic research and disease modeling, and a promise for finding and screening more effective and safe drugs, besides the possibility of use in regenerative medicine. This work obtained a model for study and treatment of SCA using iPSC. Then, episomal vectors were used for reprogramming peripheral blood mononuclear cells to obtain integration-free iPSC. Cells were collected from patients treated with hydroxyurea and without treatment. The iPSCP Bscd lines were characterized for pluripotent and differentiation potential. The iPSC lines were differentiated into HSC, so that we obtained a dynamic and efficient protocol of CD34+CD45+ cells production. We offer a valuable tool for a better understanding of how SCA occurs, in addition to making possible the development of more effective drugs and treatments and providing better understanding of widely used treatments, such as hydroxyurea.

## 1. Introduction

Sickle cell anemia (SCA) is one of the most common hereditary hematological diseases in the world, reaching a significant proportion of the population in different countries. It is particularly common among people whose ancestors came from sub-Saharan Africa, South America, Cuba, Central America, Saudi Arabia, India, and Mediterranean countries such as Turkey, Greece, and Italy. In the United States, the disease affects about 72,000 people and occurs in about 1 in 500 African Americans born and every 1 in 1000–1400 Hispanic Americans born [1] (WHO, http://www.who.int/). In Latin America, 8% of Afro-descendents have the mutated gene, which occurs in 1 every 1000–4000 Hispanic-American births [2]. In Brazil, it is the most prevalent hereditary disease, with about 1 carrier per 1500 born, with 700 to 1000 new cases per year; it is estimated that there are more than 2 million HbS gene carriers and more than 3000 affected with the homozygous form (Ministry of Health, http://www.saude.gov.br). Described in 1910 by Herrick [3], SCA is a hereditary, monogenic, autosomal codominant inheritance, resulting from a recessive mutation in the *β*-globin gene, located in the chromosomal region 11p15.5. Replacement of a single nucleotide changes the codon of the sixth amino acid, from glutamic acid to valine (G**A**G → G**T**G: Glu6Val). This mutation causes an abnormal hemoglobin, called hemoglobin S (HbS) [4, 5]. It manifests with injuries in several organs, causing high morbidity and mortality, approximately 3.4% of deaths in affected children under 5 years [6]. Infections are the main cause of morbidity and mortality in SCA, particularly in childhood [7].

Although monogenic, being defined by a single change in a specific nucleotide of genomic DNA, the clinical manifestations of SCA are extremely variable among individuals; while some patients have a very serious condition and are subject to numerous complications and frequent hospitalizations, with a high mortality rate, others present a more benign, in some cases, almost asymptomatic evolution. Hereditary and acquired factors contribute to this clinical variability, such as fetal hemoglobin (HbF) levels and socioeconomic status. However, these factors relate to more severe forms or not but do not explain these variations in their entirety.

Some available treatments include, for example, the use of hydroxyurea, the first drug approved for the treatment of sickle cell anemia. This chemotherapeutic agent acts by reactivating the production of fetal hemoglobin (HbF), a form present in newborns, and recent studies show an increase in patient survival [8]. However, the use of this drug, which only controls the symptoms, can cause side effects such as myelosuppression, particularly the granulocytic series, and the possibility of increasing the risk of tumor development, which increases even more with the long time of use [9]. The only potentially curative treatment for sickle cell anemia is hematopoietic stem cell (HSC) transplantation, with the goal of replacing the patient's bone marrow with cells without the mutation [10]. However, this is a risky procedure with high morbidity and mortality, which presents the risk of developing graft versus host disease (GVHD), making it recommended only for the most severe cases.

Induced pluripotent stem cell (iPSC) can originate any cell type and represent an alternative source to derive patient-specific blood cells. IPSC technology emerged in 2006 as a powerful tool for basic research, tissue differentiation research, and disease modeling and a promise for future clinical applications to find and screen new, more effective and safe drugs, besides the possibility of use in regenerative medicine, in the production of patient-specific cells for cell therapy. As iPSC can be expanded indefinitely in vitro and can differentiate into hematopoietic cells with blood reconstitution capacity [11, 12], this technology provides a great chance to improve the results of bone marrow transplantation, since it provides an unlimited number of immunologically compatible HSCs [13, 14]. Patients with monogenic hematological diseases, such as SCA, would be the major beneficiaries of the bone marrow transplantation procedure based on iPSC. The treatment of SCA in murine model using iPSC corrected by gene editing provided a proof of concept that the clinical application of iPSC to treat genetic blood disorders is possible.

Although iPSCs were initially obtained from fibroblasts using retroviral vectors, multiple strategies were developed for generation of iPSC without integration from fibroblasts and other cell types, including blood cells [15, 16]. The most used cells in the reprogramming have been the skin fibroblasts with the use of viral vectors [17–20], whose protocol to obtain iPSC is very well defined. However, the need for skin biopsies and the expansion of these cells by multiple in vitro passages represent a challenge that must be overcome to make iPSC technology widely applicable. Evidence obtained from several recent studies indicates that other human somatic cells can be used to generate human iPSCs, such as keratinocytes, hepatocytes, neural progenitor cells, and adipose tissue stem cells, among others [21]; but obtaining large amounts of these cell types is still a very time-consuming procedure. An easily accessible tissue that can be obtained using less invasive procedures is peripheral blood. Circulating T cells can be easily obtained from peripheral blood and can be induced to proliferate by stimulation with cytokines [22]. However, the use of these cells for reprogramming should be avoided because they are cells that do not have their genome intact, due to somatic rearrangements, creating prereorganized V(D)J DNA segments. Recently, episomal plasmid-based protocols have demonstrated the generation of integration-free iPSC from human bone marrow and umbilical cord cells [23, 24]. Although reprogramming of peripheral blood cells has a lower efficiency [23], it represents a much more affordable and abundant source of patient cells for reprogramming without the need for extensive maintenance in culture [25].

The iPSC has been used as an experimental platform for in vitro disease model. Several groups have demonstrated that cell types related to a disease are differentiated from iPSC and can faithfully reproduce the disease phenotypes [26–29]. The discovery of iPSCs has opened up a new possibility for the development of in vitro disease models in humans for the investigation of pathophysiology and for aiding drug development [30–34], and these, in the short term, are the most important aspects of this technology. The potential use of iPSC as a treatment of diseases has been proposed and tested in animal models in vitro and in vivo with promising results [35–37].

With the iPSC field progressing so rapidly, the next challenge will be to demonstrate the functional utility of iPSC-derived cells in preclinical models of various human diseases and eventually move that technology to the clinic [38]. In recent years, great progress has been made in the development of hematopoietic differentiation systems and the production of major blood cell types from human pluripotent stem cells (hPSCs) [14]. Even so, the generation of hematopoietic cells with robust and long-term reconstitution potential remains a major challenge.

In this study, we explored the use of a nonintegrating episomal vetor to reprogram adult blood cells from sickle cell anemia patients and establish a study and treatment model for SCA, through the establishment of an efficient protocol for obtaining hematopoietic progenitor cells. iPSC generated can be used in the future to correct the *β*-globin gene mutation and differentiate into hematopoietic cells. The methodology developed in this study has potential applications in iPSC banking and in disease modeling for other genetic disease, so in the future, these iPSCs could be used in regenerative medicine. Establishing an in vitro model of the disease allows a better understanding of how the disease occurs, the possible causes of the clinical differences demonstrated by the patients affected, and the development of new tests and more effective treatments against the disease. IPSCs, while still unsuitable for clinical use, have the potential to revolutionize the way we study human development, generate life-threatening disease models, and eventually how we treat patients.

## 2. Materials and Methods

### 2.1. Cell Preparation

The cells reprogrammed in this study were obtained through peripheral blood collection of sickle cell anemia patients from blood center of Ribeirao Preto, after acceptance and signature of the informed consent. The protocol was analyzed and approved by the local ethics committee (number: 486.426). The inclusion criteria were age over 18 years, homozygous for the S allele, and absence of treatment with hydroxyurea. About 12 mL of blood was collected from a superficial vein of the arm, after antisepsis of the puncture site, by a qualified professional. Peripheral blood MNCs were separated by Ficoll-Hypaque Premium (GE Healthcare) density gradient, according to the manufacturer's instructions and as described [39, 40]. The cells were freezed at a concentration of 1 × 10^7^ cells per vial in fetal bovine serum (Hyclone) supplemented with 20% dimethyl sulfoxide [DMSO (Sigma-Aldrich)]. Of the MNC obtained, 1-2 × 10^6^ was cultured with StemSpan™ medium (STEMCELL Technologies) supplemented with the following cytokines: 100 ng/mL stem cell factor (SCF), 10 ng/mL IL-3, 2 U/mL erythropoietin (EPO), 40 ng/mL insulin-like growth factor 1 (IGF-1) (all from Peprotech), and 1 *μ*g/mL dexamethasone (Sigma-Aldrich). The cells were counted, and the medium was changed every 3 days. The day 12 and day 14 cultured cells were used for reprogramming. This culture condition favors the enrichment erythroblastic population and does not support the growth of the lymphoid population. After 3 days of thawing and after 12 days of expansion, the cells were evaluated for lymphoid cell markers and erythroblast/erythroid cell markers by flow cytometry.

### 2.2. Generation of iPSC with Episomal Vectors and Culture of iPSC

The MNCs cultured in MNC medium for 12–14 days were used for the reprogramming process by the transfection of the episomal plasmids, pEB-C5, expressing five factors (OCT4, SOX2, KLF4, C-MYC, and LIN28), and pEB-Tg, expressing SV40 large T antigen, in 2.5 × 10^6^ cells [33]. These episomal plasmids are available from Addgene (plasmid numbers 28213 [pEB-C5] and 28220 [pEB-Tg]). The transfection was performed using the Lonza human CD34+ cell nucleofector kit (Lonza) on the Amaxa nucleofector II device (Lonza), with a total of 2.5 × 10^6^ cells at a cell suspension of 100 *μ*L combined with 8 *μ*g of DNA of the pEB-C5 plasmid (≥1 *μ*g/*μ*L) and 2 *μ*g of DNA of the pEB-Tg plasmid (≥1 *μ*g/*μ*L). After transfection, the cells were plated back in the expansion medium in one well of a 12-well plate to allow recovery and were incubated at 37°C, 5% CO_2_, 5% O_2_. Two days later, they were plated onto standard plates coated with feeder cells, with the culture medium changing to ESC medium [20% knockout serum replacement, 2 mM L-glutamine, 0.1 mM nonessential amino acids, 0.1 mM *β*-mercaptoethanol, 50 U/mL penicillin, 50 *μ*g/mL streptomycin, and 10 ng/mL basic fibroblast growth factor (bFGF) in Knockout Dulbecco's modified Eagle's medium (Knockout DMEM) (all from Gibco™)] the following day. At this point, we added sodium butyrate (NaB) (0.25 mM) (Sigma-Aldrich) to the cultures to enhance iPSC derivation. The medium was changed every other day, until small colonies begin to appear, when the medium started being changed every day. On day 9 after transfection, the cells started to being fed with conditioned medium (CM). The use of CM is necessary at this time to maintain colony growth. For the production of CM, we used a 12-well plate with mitotically inactivated MEFs plated at a density of ~100,000 cells per well with ESC medium. Every day, we collected and replaced the ESC medium. CM was collected daily for 4-5 days, as long as the MEF morphology was acceptable.

During days 9–11 after transfection, colonies with ESC-like morphology start to become visible and, during days 14-15, large colonies were picked, expanded, and examined for pluripotency markers. The colonies were picked manually under an inverted microscope after treatment with 0.5 mM EDTA. After the first picking, the human iPSCs were maintained and expanded in mTeSR™1 medium (STEMCELL Technologies) on matrix Matrigel™ (Corning). The expanded colonies were evaluated for pluripotency and self-renewal characteristics and differentiation potential.

### 2.3. RNA Isolation, Reverse Transcription, and qPCR

Total RNA from iPSC, from MNC, and from ESC was purified with Trizol reagent (Invitrogen) and treated with DNase using the RNeasy mini kit (Qiagen, Hilden, Germany). Two micrograms of total RNA was used for reverse transcription reaction using the high-capacity cDNA reverse transcription kit (Applied Biosystems) according to the manufacturer's instructions. Quantitative polymerase chain reaction (qPCR) was performed by TaqMan (Applied Biosystems) and analyzed with the 7500 real-time PCR system (Applied Biosystems). All experiments were performed in duplicate, and a nontemplate control (lacking cDNA template) was included in each assay. Gene expression was normalized relative to that from endogenous gene glyceraldehyde 3-phosphate dehydrogenase (GAPDH) and *β*-actin (Applied Biosystems). The primer sets used for detecting ESC marker gene expression were the pluripotency markers OCT4, SOX2, and NANOG (Applied Biosystems).

### 2.4. Immunocytochemistry Assay

Reprogrammed cells were fixed in 4% paraformaldehyde (Merck) for 20 minutes at room temperature, washed twice with phosphate-buffered saline, and then permeabilized with 0.2% Triton X-100 (Sigma-Aldrich) for 1 hour at room temperature. The cells were blocked for 1 hour with 5% bovine serum albumin and 2% goat serum in phosphate-buffered saline solution. Samples were incubated at room temperature for 1 hour with primary antibodies against antistage-specific embryonic antigen 4 (SSEA4) (STEMCELL Technologies), octamer-binding transcription factor 4 (OCT4) (Chemicon), sex-determining region Y-box 2 (SOX2), and NANOG homeobox (NANOG). The secondary antibodies used were AlexaFluor 564 mouse antibodies (Molecular Probes, Invitrogen), incubated at room temperature for 1 hour. The nucleus was stained with 4′,6-diamidino-2-phenylindole (VYSIS). Cells were visualized using a confocal laser scanning microscope (LSM 710; Carl Zeiss) with objective lens ×63 in oil immersion, having a numerical aperture of 1.4. Argon laser of 590 nm was used to excite the surface marked with secondary antibodies, and the emission was measured at 617 nm. Image analysis and colocalization studies were carried out using the ZEN 2008 system confocal software (ZEN, version 2.5).

### 2.5. Flow Cytometry

The immunophenotypic characterization was perfomed by flow cytometry using the following monoclonal antibodies: NANOG-fluorescein isothiocyanate (FITC), OCT3,4-phycoerythrin (PE), and SOX2-PE (Pharmingen).

Cells were harvested with StemPro® Accuttase® Cell Dissociation Reagent (Gibco) and were incubated with the antibodies following the manufacturer's instructions. Nonspecific immunoglobulin G of the corresponding class served as the negative control. Cell suspensions were analyzed on FACSort flow cytometer (Becton-Dickinson) using CellQuest software.

### 2.6. Alkaline Phosphatase Assay

Alkaline phosphatase (AP) staining was performed with the AP leukocyte alkaline phosphatase kit (Sigma-Aldrich) according to the manufacturer's instructions. High levels of AP expression indicate undifferentiated cells with self-renewal potential. Reddish pink-stained cells were classified as positive for AP expression [41].

### 2.7. In Vitro Differentiation of Human Reprogrammed Cells

For spontaneous differentiation through embryoid body (EB) formation, reprogrammed cells were harvested by 0.5 mM EDTA treatment. The cell clumps were transferred to a 6-well low adhesion plate in mTeSR1 medium (STEMCELL Technologies). After 5 days in suspension culture, EBs were transferred to a 6-well gelatin-coated plates (0.1%) and cultured in DMEM suplemmented with 10% FBS, 1% antibiotic-antimycotic, and 2 mM L-glutamine (all from Gibco) for 15 additional days, changing medium every other day. This condition allows EB cells to spontaneously differentiate in vitro. After 15 days, the cells were collected for RNA extraction and qPCR analyses. For detecting the diferentiation potential in the formed EB, we used Nestin, alpha-smooth muscle actin (*α*-SMA) and alpha-fetoprotein (AFP) (Applied biosystems) markers for the identification of cells from ectoderm, mesoderm, and endoderm, respectively.

### 2.8. In Vivo Multilineage Differentiation

The evaluation of the differentiation potential was performed in vivo by the teratoma formation assay in immunodeficient mice. Approximately 2 × 10^6^ iPSC clumps were subcutaneously injected into NOD/SCID Gamma mice. After approximately 10–12 weeks, mice were sacrificed and tissues were analyzed for tumor formation. The histological processing of the teratoma, preserved in 10% formalin for 24 hours, comprised a dehydration battery followed by clarification and immersion and inclusion in paraffin. The sections were fixed, clarified, and dehydrated for staining with hematoxylin and eosin for morphological analysis. The sections were subjected to a new dehydration and clarification, and the slides were set up with Entellan® (Merck Millipore) adhesive gel.

The sections for immunohistochemistry assay were incubated at 50°C overnight. For immunostaining, the sections passed through a battery of clarification and dehydration. The antigen retrieval was performed with citrate buffer (10 mM, pH 6), and peroxidase blockade was performed with 3% H_2_O_2_ diluted in methanol. Blocking of nonspecific binding was performed with PBS-0.25% Tween +5% BSA. The antibody was applied, diluted in 0.1% PBS-Triton + 1% BSA, and incubated overnight. The biotinylated secondary antibody [Universal Dako Cytomation Labelled Streptavidin-Biotin System, Horseradish Peroxidase (Dako)] was applied and incubated, followed by applying the tertiary antibody streptavidin (supplied with the kit described above). The DAB (supplied with the kit described above) was diluted and applied, followed by the application of hematoxylin. The sections were subjected to a new dehydration and clarification, and the slides were set up with Entellan (Merck Millipore) adhesive gel. Sections were analyzed by immersion microscopy.

### 2.9. Assessment of Pluripotency and Prediction of Differentiation Potential

We used the TaqMan hPSC Scorecard (Applied Biosystems) methodology, which contains specially formulated gene expression assays for the evalution of iPSC and CTE, to confirm the self-renewal potential and predict the differentiation potential of the lineages generated. Total RNA from iPSC, from MNC, and from ESC was purified with Trizol reagent (Invitrogen) and treated with DNase using the RNeasy mini kit (Qiagen). One microgram of total RNA was used for reverse transcription reaction using the high-capacity cDNA reverse transcription kit (Applied Biosystems) according to the manufacturer's instructions, and the amplification conditions are 25°C for 10 minutes, 37°C for 120 minutes, and 85°C for 5 minutes. The prepared cDNA samples were diluted with nuclease-free water and 2X TaqMan Fast Advanced Master Mix, according to the manufacturer's instructions. The mixture was distributed on the 96-well plate and, after sealing and centrifugation, the plate was cycled in equipment suitable for fast thermocycling following the amplification parameters: 1 cycle at 50°C for 20 seconds and 40 cycles at 95°C for 1 second and 60°C for 20 seconds. Gene expression data were analyzed using the hPSC Scorecard analysis software available online (http://lifetechnologies.com/scorecarddata).

### 2.10. Screening for Occurrences of Spontaneous Integrations

To evaluate the iPSC DNA for the presence of spontaneous integrations of the episomal plasmids used in the generation of the cells, we performed the screening through PCR with two sets of primers designed from sequences pre-established in literature [42] and synthesized for amplification and identification of each one of the vectors used in cell generation. The analyzed cells were iPSC generated at different passages, which were dissociated by enzymatic treatment with the StemPro Accutase® dissociation reagent (Gibco). The cell suspension was collected, washed with PBS (Gibco), and used for DNA extraction with Dneasy Blood & Tissue kit (Qiagen), according to the manufacturer's instructions. After extraction, the DNA was quantified in spectrophotometer (Nanodrop-Spectrophotometer ND-1000) at 260 nm.

As positive control, we used expanded MNCs which were nucleofected with the two vectors, pEB-C5 and pEB-Tg, as described in item above. Transfected cells were incubated for for 48 hours and then were collected for DNA extraction as described above. The set of primers are shown in Table 1.

For the amplification reaction, we used 10 pmols of each primer, 1 *μ*L of 10 mM dNTP, 5 *μ*L of 10× buffer (200 mM Tris, pH 8.4, 500 mM KCl) (Invitrogen), 1.5 *μ*L of 50 mM MgCl_2_ (Invitrogen), and 0.2 *μ*L of 5 U/*μ*L Taq DNA Polymerase (Invitrogen) in a final volume of 50 *μ*L. The reactions were thermocycled in MyCycler® Thermal Cycler (Bio-Rad) with the following amplification program: 94°C for 10 minutes; 35 cycles of 94°C for 45 seconds, Ta appropriate for each set of primer for 45 seconds and 72°C for 90 seconds; a final extension of 72°C for 7 minutes. The amplification evaluation was performed by agarose gel electrophoresis 1.5% stained with ethidium bromide.

### 2.11. Screening for Glu6Val Mutation

For the molecular identification of the mutation responsible for the sickle cell phenotype, the generated iPSC lines were subjected to amplification of the *β*-globin gene (HBB) and subsequently submitted to sequencing. The cells used were the iPSC lines generated and a negative control of the reaction without any sample. The primers used in the amplification reactions were HBB forward (5′-GAAGAGCCAAGGACAGGTAC-3′) and HBB reverse (5′-CAACTTCATCCACGTTCACC-3′).

For the amplification reaction of the HBB gene, we used 0.06 *μ*g of DNA, 1 *μ*L dNTP 10 mmol/L (Invitrogen), HBB forward 10 pmols, HBB reverse 10 pmols, 5 *μ*L of 10× enzyme buffer (200 mmol/L Tris, pH 8.4, 500 mmol/L KCl) (Invitrogen), 2 *μ*L of MgCl2 (50 mmol/L) (Invitrogen), and 0.2 *μ*L of Taq DNA polymerase (5 U/*μ*L) (Invitrogen) in a final volume of 50 *μ*L. This reaction was thermocycled in MyCycler Thermal Cycler (Bio-Rad) with an amplification program of 94°C for 10 minutes; 35 cycles of 94°C for 45 seconds, 55°C for 45 seconds, and 72°C for 90 seconds; and a final extension of 72°C for 7 minutes.

To verify the amplification of the HBB gene, 1.5% agarose gel electrophoresis stained with ethidium bromide was performed.

The amplified samples were submitted to sequencing of the HBB gene to locate the Glu6Val mutation responsible for the sickle cell phenotype. For the sequencing of a sample of each generated iPSC line, a new PCR reaction was performed under the same conditions described above. The product of this first PCR reaction for sequencing was subjected to a second reaction in which 1 *μ*l of this was added to 2 *μ*l of Big Dye Terminator v3.1 Cycle Sequencing Kit (Applied Biosystems) and 5 pmols of primer HBB forward or 5 pmols of primer HBB reverse, in a final volume of 10 *μ*L. These reactions, made in triplicate each, were thermocycled in the MyCycler Thermal Cycler (Bio-Rad), using the amplification program of 95°C for 1 minute, 25 cycles of 95°C for 10 seconds, 51°C for 5 seconds, and 60°C for 4 minutes. Then, a precipitation of the PCR product following the ABI/Isopropanol (MERCK) protocol was performed, for the elimination of unincorporated dNTPs, ddNTPs, and enzymes. The samples were submitted to electrophoresis in the automatic sequencer ABI 3500XL Genetic analyzer (capillary electrophoresis system), using Sanger's method, based on chain termination chemistry with dideoxynucleotides (ddNTPs). In the form of an electropherogram, the DNA sequences generated were directly sent to a computer connected to the sequencing apparatus. These electropherograms were interpreted by the software DNA Sequencing Analyzer 4.0 and converted into DNA sequences, which were later analyzed using the ChromasPro program.

### 2.12. Hematopoietic Differentiation

The iPSCs obtained were differentiated into hematopoietic progenitors/hematopoietic stem cells (HSC) using a combination of available protocols [43, 44]. Initially, iPSCs underwent an adaptation step to enzymatic picking with StemPro Accutase dissociation reagent (Gibco) supplemented with ROCk inhibitor (Tocris) at 37°C. The cell suspension was washed with PBS (Gibco), counted using Neubauer's chamber and 0.4% Trypan Blue solution (Gibco), and plated at high cell concentration. This culture was maintained for at least 4-5 passages until identification of complete adaptation, that is, when cells after individualization by treatment with dissociation reagent are again grouped into colonies.

After adaptation, the cells were again dissociated and counted, and approximately 4000 cells were plated per well in a low-adhesion 96-well plate in STEMdiff™ APEL™ (STEMCELL Technologies) differentiation medium supplemented with the BMP4 (10 ng/mL) (Peprotech) and bFGF (10 ng/mL) (Peprotech) cytokines, in addition to the ROCK inhibitor (10 mM) (Tocris), in a final volume of 50 *μ*L per well. The plates were centrifuged at 300 ×g for 5 minutes to form aggregates and incubated at 37°C and 5% CO2. The experiment was conducted for 14 days, with fresh medium additions every 3 days. The fresh medium was supplemented with the cytokines BMP4 (10 ng/mL) (Peprotech), bFGF (10 ng/mL) (Peprotech), VEGF (10–20 ng/mL) (Peprotech), and SCF (50–100 ng/mL) (Peprotech). On differentiation induction days 0, 4, 8, 11, and 14, cells were collected for evaluation of pluripotency and hematopoietic progenitors' markers by flow cytometry. For the analyses, the EBs were collected, centrifuged at 160 ×g for 4 minutes, and dissociated with StemPro Accutase (Gibco) dissociation reagent at 37°C for about 15–20 minutes. The dissociated cells were homogenized using 22-gauge needle, centrifuged at 250 ×g for 4 minutes, and resuspended in 1 mL PBS (Gibco), and an aliquot was took out for counting.

On day 11 of hematopoietic induction, EBs were also collected for colony-forming cell (CFC) assay in methylcellulose. For the assay, 2 × 10^5^ cells (140 *μ*L) were homogenized with 3 mL of methylcellulose medium with recombinant cytokines [MethoCult H4434 classic (StemCell)]. The mixture was distributed in 3 plates of 35 mm, 1 mL/plate, which were incubated at 37°C for about 15 days, when they could be analyzed for colony formation.

## 3. Results

### 3.1. Reprogramming Mononuclear Cells into iPSC by Episomal Vectors

**Figure 1:**
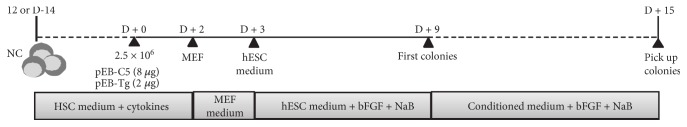
Scheme of the reprogramming protocol used in the generation of iPSCs from adult MNCs. After nine days of nucleofection, the first colonies were visible and after approximately 15 days, the first picking was performed.

MNCs were isolated from six patients with sickle cell anemia (SCA), after diagnostic confirmation of SCA, four males and two females, three in treatment with hydroxyurea (HU) and three who were not under treatment with HU, ranging in age from 19 to 32 years. The Ficoll-Hypaque processing of the samples resulted in a yield of about 1–3 × 10^7^ cells. Cells were frozen at a concentration of 5 × 10^6^ cells/vial; each patient sample generated approximately 2–6 vials.  Figure 1 shows a schedule of the reprogramming process used to generate iPSC from MNC.

**Figure 2:**
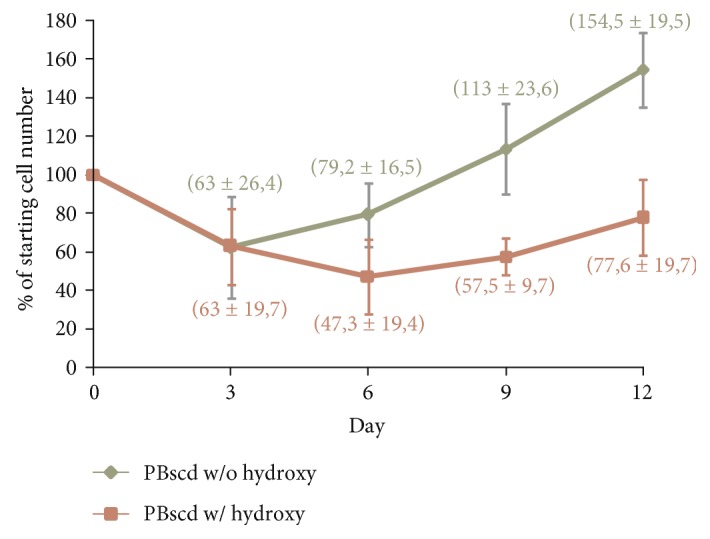
Graph of representative cell growth data showing percentage of cell number during expansion in a culture for nonlymphoid cell enrichment and lymphoid cell depletion. Data were expressed as mean ± standard deviation. w/o hydroxy: without hydroxyurea; w/ hydroxy: with hydroxyurea.

**Figure 3:**
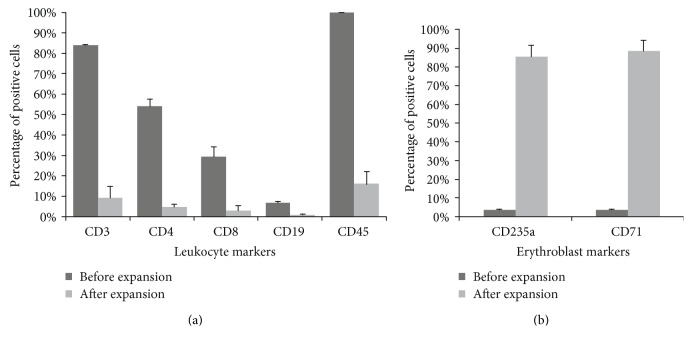
Graphs of representative data of flow cytometry, analysis of markers before and after culturing in specific medium for lymphoid cell depletion and erythroblast/erythroid lineage induction. (a) Percentage of positive cells for leukocyte markers. (b) Percentage of positive cells for erythroblast/erythroid markers.

The MNCs were expanded in StemSpan medium containing a combination of SCF, IL-3, IGF-1, EPO, and dexamethasone for 12–14 days for lymphocyte depletion and erithroblast expansion. This expansion was aimed at reducing the risks of reprogramming a cell of the lymphoid lineage. The expansion comprised about 12–14 days, with medium changes and counts every 3 days. For the cells to maintain adequate growth and to have no limitation of their growth, they were maintained at a concentration of 2 × 10^6^ cells/mL. Cell growth was assessed throughout the expansion process. MNCs from patients treated with HU showed lower cellular expansion than those from untreated patients (Figure 2). Patient samples not treated with HU showed a cell growth of 55% (±19.5) relative to the initial cell percentage. In cells of patients treated with HU, not even the initial cell population was reached after consecutive falls on days 3 and 6 of culture in specific medium. After 3 days of MNCs thawing and after 12 days of expansion in specific medium, cells were evaluated for lymphoid cell markers and erythroblast/erythroid cell markers by flow cytometry (Figure 3). It was found that a large population resemble cells of the erythroblast/erythroid line, expressing high levels of CD71 (88.5% ± 5.5%) and CD235a (85.3% ± 6.3%) that are not found at the beginning of the expansion (CD71: 3.5% ± 0.5%, CD235a: 3.7% ± 0.17%). There was also a decrease in the cell population expressing T cell markers [CD4 (4.6% ± 1.6%) and CD8 (3% ± 2.4%)], B cells [CD19 (0.9% ± 0.2%)], and common leukocyte markers [CD45 (16% ± 6%)], when compared to the cells at the start of induction. Initial expression of these markers in the cells was high for T cells [CD4 (54% ± 3.7%) and CD8 (30% ± 5%)], B cells [CD19 (6.7% ± 0.7%)], and leukocytes [CD45 (99.8% ± 0.02%)] (data were expressed as mean ± standard deviation).

**Figure 4:**
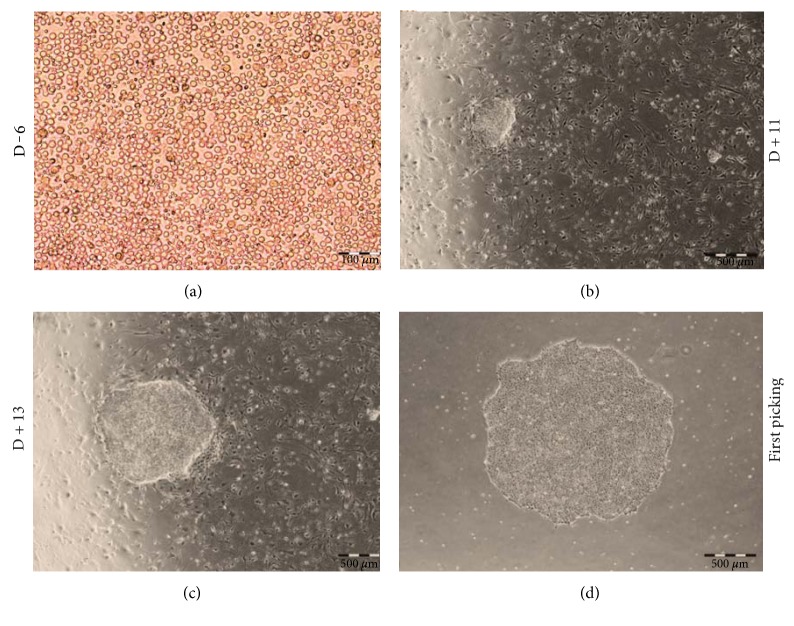
Reprogramming process of iPSC PBscd02, showing the alteration in the cell morphology, passing from individualized cells with growth in suspension to cells with growth in colonies. (a) PBMCscd02 with 6 days of expansion in specific medium for lymphoid cell depletion and nonlymphoid cell enrichment. (b) Emergence of the first colonies on MEF, 11 days after the episomal transfection. (c) Colony growth on MEF, with 13 days postnucleoporation culture, before the first peal. (d) Colony established after the first peal for matrix Matrigel. Images in phase contrast. Optical microscopy.

On day 12 or 14, the expanded cells (2.5 × 10^6^) were transfected with episomal vectors carrying mouse OCT4, SOX2, KLF4, C-MYC, LIN28, and SV40 large T. The first colonies were observed aproximately 9 days after transfection and picked up by days 14-15. Large changes were observed in the morphology of cells that changed from small, rounded, individualized cells that grow in suspension to epithelial cell morphology, characterized by small, juxtaposed, colony-growing ESC-like cells (Figure 4). Six patient samples were submitted to erythroblast expansion followed by transfection with plasmids for reprogramming. From these samples, we obtained 4 pluripotent colonies, 3 samples from patients not being treated with HU and only 1 patient sample being treated with HU. The only patient sample on treatment with HU that was reprogrammed (PBscd08^HU^) had a low number of colonies (only 4), which impacted in reprogramming efficiency (0.0011%) (Table 2). The other two samples from patients on HU treatment (PBscd06^HU^ and PBscd07^HU^) were submitted to erythroblast expansion and transfection; however, after extensive culture in ESC medium, they did not present colony formation. Table 2 shows the reprogramming efficiency of MNCs by episomal vectors and summarizes all details of the 6 experiments. Regarding reprogramming, we found that cells derived from patients not treated with hydroxyurea had an 11-fold improvement in the generation of colonies compared to cells derived from treated patients.

The lineage generated from MNCs from patients on treatment with HU, iPSC PBscd08^HU^ generated 4 colonies that were used to clonal picking due to the low number of them. For the isolation of the clones, one colony at a time was picked and transferred to a 24-well plate, one colony per well, previously covered with matrix and with mTeSR1 medium. Three clones of the iPSC line PBscd08^HU^, clones 02, 03, and 04, were generated after expansion. These clones, as well as the colonies of the other lines obtained, were expanded in matrix and after successive passages were characterized for the pluripotent and differentiation potential.

### 3.2. Characterization of Integration-Free iPSC Derived from Adult PB

**Figure 5:**
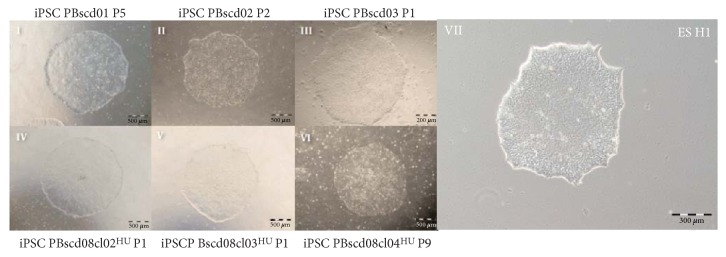
Colonies of iPSC PBscd from all experiments, demonstrating the ES-like morphology, characterized by growth in colonies of small and juxtaposed cells. Phase contrast images. Optical microscopy.

PBscd iPSCs, generated as described above, robustly proliferated under human iPSC/ESC culture conditions, remaining undifferentiatiated and morphologically alike for more than 100 passages. PBscd iPSC colonies showed a morphology of juxtaposed cells of growth in colonies, characteristic of human pluripotent stem cells (Figure 5). All the samples used to reprogram show colonies with ESC-like morphology.

The complete characterization was performed for the first lineage generated, iPSC PBscd01, to certify that the method could generate pluripotent colonies. This characterization included the evaluation of the pluripotency by flow cytometry, qRT-PCR, immunocytochemistry, alkaline phosphatase assay, assessment of the differentiation potential in vitro through the formation of embryoid bodies, differentiation potential prediction by the TaqMan hPSC Scorecard methodology, in addition to the teratoma formation assay for the assessment of the differentiation potential in vivo, and evaluation of the sections by immunohistochemistry and HE. For the following lines, the pluripotent potential was characterized by evaluation of the markers by flow cytometry and the characterization of the potential of differentiation was performed through the formation of teratomas.  Table 3 summarizes the characterization made with each iPSC line.

#### 3.2.1. Characterization of Pluripotent Potential

**Figure 6:**
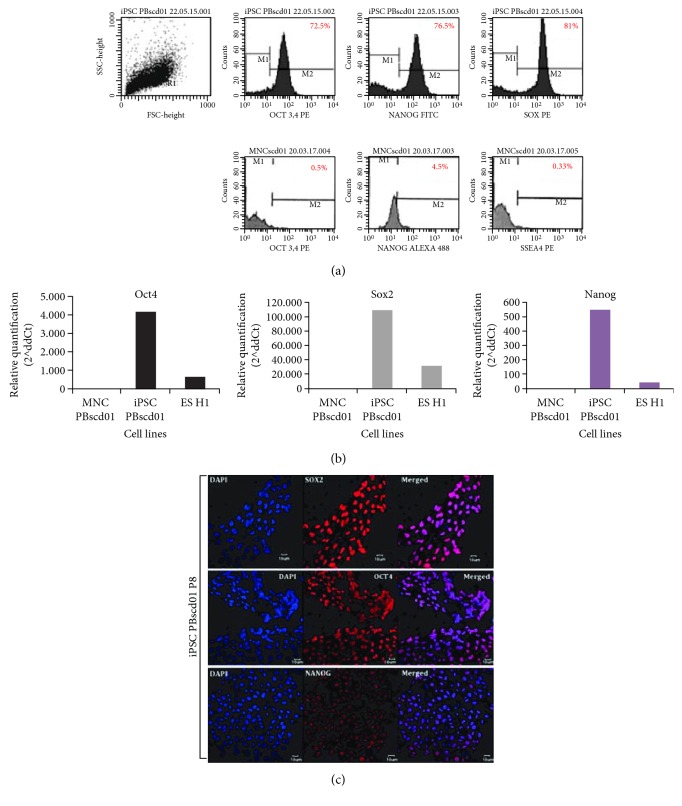
Characterization of iPSC PBscd01 and confirmation of pluripotency. (a) Flow cytometry analyses of OCT4, NANOG, and SOX2 markers in iPSC PBscd01 and OCT4, NANOG, and SSEA-4 markers in the parental cell (MNCscd01). (b) Expression of endogenous pluripotency markers confirmed by qPCR after seven passages. Quantification of the relative expression of OCT4, SOX2, and NANOG genes. Individual reactions of qPCR were normalized against internal controls (GAPDH and *β*-actin) and plotted against the level of parental cell expression (MNC PBscd01). (c) Immunostaining of the colonies of iPSC PBscd01 showing the expression of pluripotency markers OCT4, SOX2, and NANOG. Nuclei were stained with DAPI (blue). Confocal microscopy.

**Figure 7:**
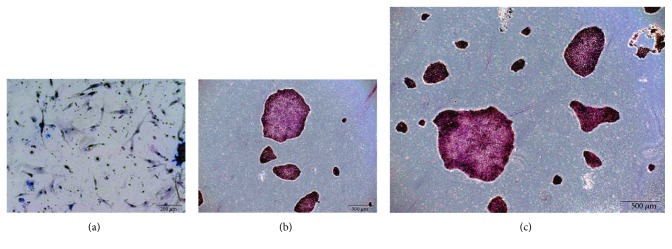
IPSC colonies PBscd01 positive for alkaline phosphatase (AP) and negative control fixed and stained, but not positive for the enzyme. (a) Fibroblasts, used as negative control, showing that they did not undergo the marking of alkaline phosphatase, confirming that they came from somatic cells, already differentiated. (b, c) IPSC PBscd01 positive for alkaline phosphatase, indicating that they are undifferentiated cells with self-renewal potential. Phase contrast images. Optical microscopy.

The iPSC PBscd01 flow cytometry characterization showed that approximately 73% of the cells are OCT4 positive, 77% of the cells express NANOG, and 81% are positive for SOX2 (Figure 6(a)). The MNCscd01 flow cytometry characterization with pluripotency markers showed that the labelling is specific to iPSC (Figure 6(a)). qRT-PCR assay was used to quantify the expression of the pluripotent genes OCT4, SOX2, and NANOG. The iPSC PBscd01 express 5000, 120,000, and 600 times more OCT4, SOX2, and Nanog, respectively, than the parental line MNCscd01, and even higher levels of expression than those observed in the hESC samples (Figure 6(b)). Immunostaining of iPSC PBscd01 colonies also showed expression of pluripotency markers OCT4, SOX2, and NANOG (Figure 6(c)). The iPSC PBscd01 also demonstrated high expression of alkaline phosphatase, characterized by reddish pink or purplish red cells when fixed and stained, indicating that they are undifferentiated cells with self-renewal potential (Figures 7(b) and 7(c)), different from the somatic cells and fibroblasts, used as negative control (Figure 7(a)).

**Figure 8:**
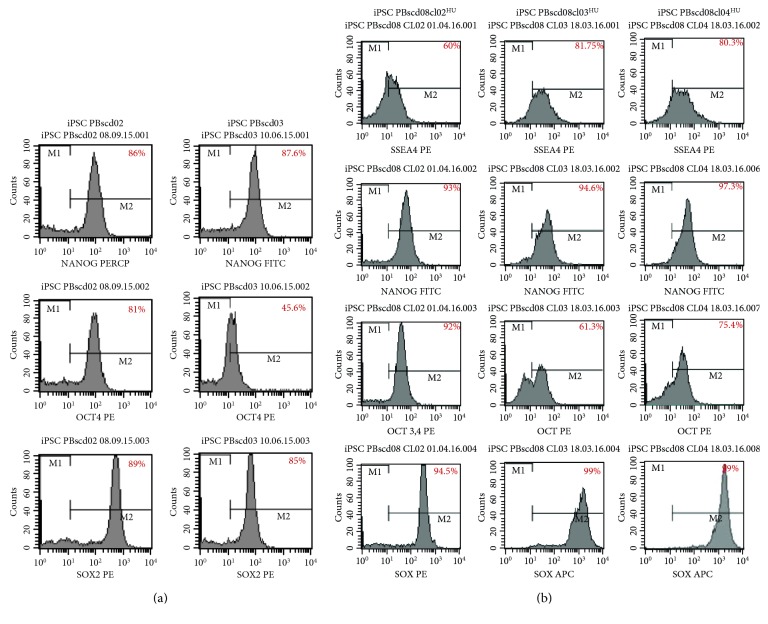
Flow cytometry analyses for pluripotency markers OCT4, SOX2, NANOG and SSEA-4 of the iPSC PBscd lines generated.

For the other generated iPSC PBscd lines (iPSC PBscd02, iPSC PBscd03, iPSC PBscd08cl02^HU^, iPSC PBscd08cl03^HU^, and iPSC PBscd08cl04^HU^), we evaluated the pluripotent potential by flow cytometry characterization of pluripotency markers OCT4, SOX2, NANOG, and SSEA-4 (Figure 8). IPSC PBscd02 showed 86% of the cells positive for NANOG, 81% positive for OCT4, and 89% positive for SOX2; iPSC PBscd03 showed positivity for NANOG in 87.6% of the cells, for OCT4 in 46% of the cells, and for SOX2 in 85% of the cells (Figure 8(a)). The clone 02 of iPSC PBscd08^HU^ showed 60% of the cells positive for the surface marker SSEA-4, 93% positive for NANOG, 92% positive for OCT4, and 94.5% positive for SOX2; the clone 03 of iPSC PBscd08^HU^ showed positivity for SSEA-4 in 81.8% of the cells, for NANOG in 94.6% of the cells, for OCT4 in 61.3% of the cells, and for SOX2 in 99% of the cells; and the clone 04 of iPSC PBscd08^HU^ showed 80.3% of the cells positive for SSEA-4, 97.3% positive for NANOG, 75.4% positive for OCT4, and 99% positive for SOX2 (Figure 8(b)). Thus, all lines showed high percentages of positive cells for pluripotency markers, confirming the pluripotent potential of these cells.

#### 3.2.2. Characterization of the Differentiation Potential

**Figure 9:**
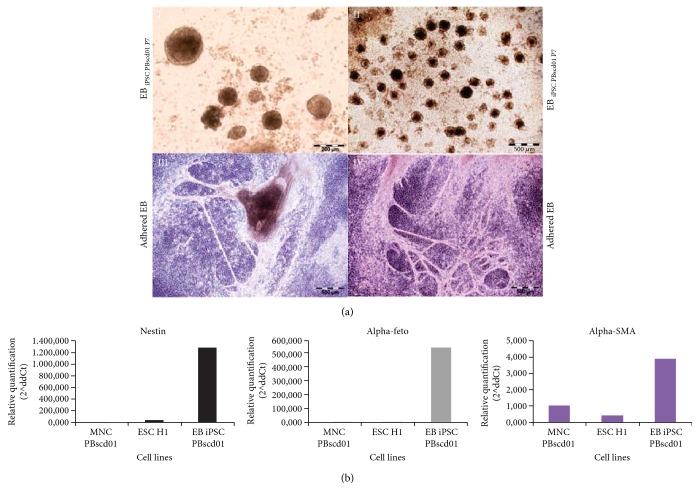
In vitro differentiation assay. (a) Confirmation of the differentiation potential of iPSC PBscd01 through spontaneous differentiation and formation of EB. Images show the in vitro morphological changes of iPSC PBscd01. (I, II) EB formed by suspension culture. (III, IV) EB after spontaneous differentiation in adherence. Phase contrast images. Optical microscopy. (b) Differentiation analyzed for the presentation of the markers of cells of the 3 germ layers, by qPCR of the EB. Individual qPCR reactions were normalized against internal controls (GAPDH and *β*-actin) and plotted against the level of parental cell expression (MNC PBscd01).

We further investigated whether PB iPSC can be induced to differentiate into cells of different lineages in culture. IPSC PBscd01 could form spherical structures called embryoid bodies (EB) when cultured under specific conditions (Figure 9(a), I-II). EBs are spherical structures formed in vitro from pluripotent cells, which harbor the development of various cell types and tissues. After the EB formation, the cells were cultured for adhesion and spontaneous differentiation (Figure 9(a), III-IV). Gene amplification by quantitative real-time PCR demonstrated that after differentiation, the cells express ectoderm marker, Nestin; the endoderm marker, AFP; and the mesoderm marker, *α*‐SMA, at higher levels than ESC H1 and MNCs (Figure 9(b)).

**Figure 10:**
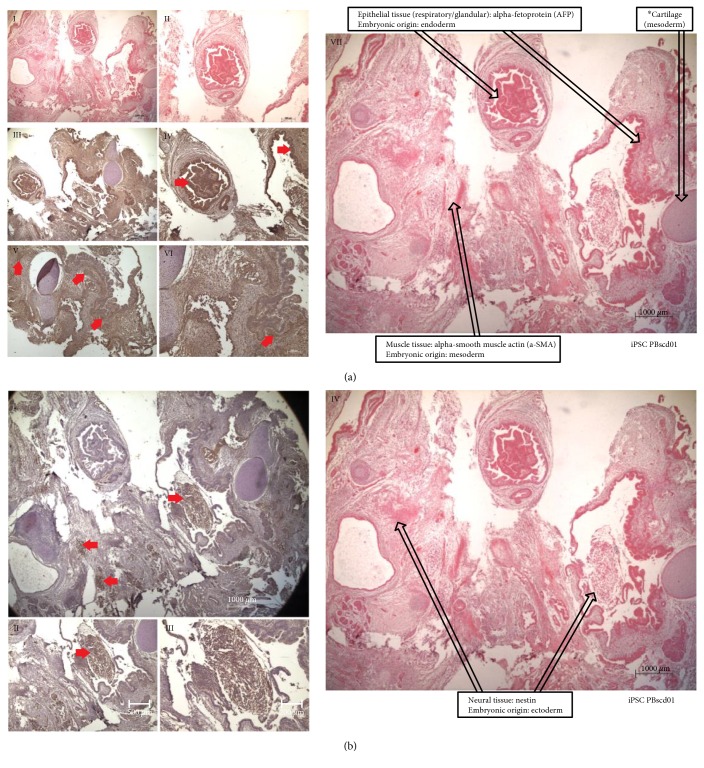
Immunohistochemistry (IHC) and staining with hematoxylin and eosin (HE) from histological sections of the iPSC PBscd01 teratoma, demonstrating the staining for the 3 germ layers. (a) (I, II, and VII) Images of HE staining, identifying respiratory and glandular epithelial tissue, muscle tissue and cartilage. (III, IV) Images demonstrating the positive staining for AFP, evidencing respiratory and glandular epithelial tissue, confirming the endodermal origin of the tissue. (V, VI) Images demonstrating the positive staining for α-SMA, evidencing muscle tissue, confirming the mesodermal origin of the tissue. (b) (I, II, and III) Images of IHQ showing positive staining for Nestin, confirming the ectodermal origin of the tissue found. (IV) Images of HE staining, identifying the neural tissue region.

**Figure 11:**
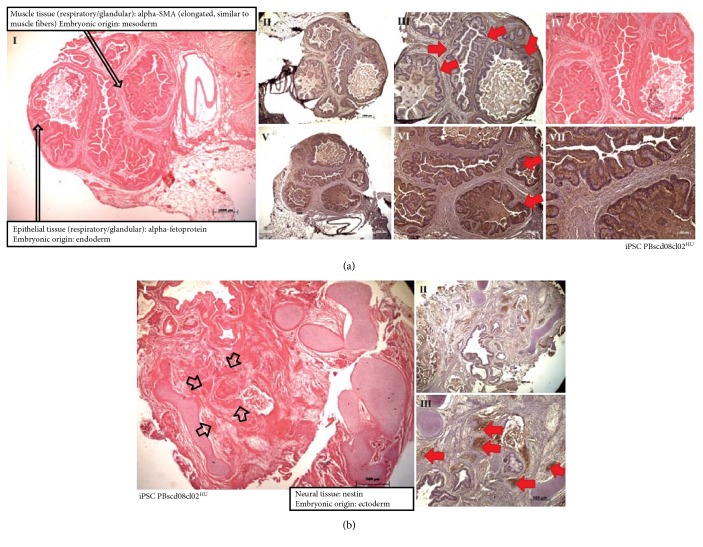
Immunohistochemistry (IHC) and staining with hematoxylin and eosin (HE) from the histological sections of the iPSC PBscd08cl02^HU^ teratoma, demonstrating the staining for the 3 germ layers. (a) (I) Image identifying muscle tissue with elongated cells like muscle fibers, of mesodermal origin, and respiratory and guandular epithelial tissue, both of endodermal origin. (II, III) Images demonstrating the positive staining for *α*-SMA, confirming the mesodermal origin. (IV) Image of HE staining. (V, VI, and VII) Images demonstrating the positive staining for AFP, confirming that they are tissues from the endodermal lineage. (b) (I) Images of HE staining, evidencing the neural tissue region. (II, III) Images of IHQ showing positive staining for Nestin, confirming the ectodermal origin of the tissue found.

**Figure 12:**
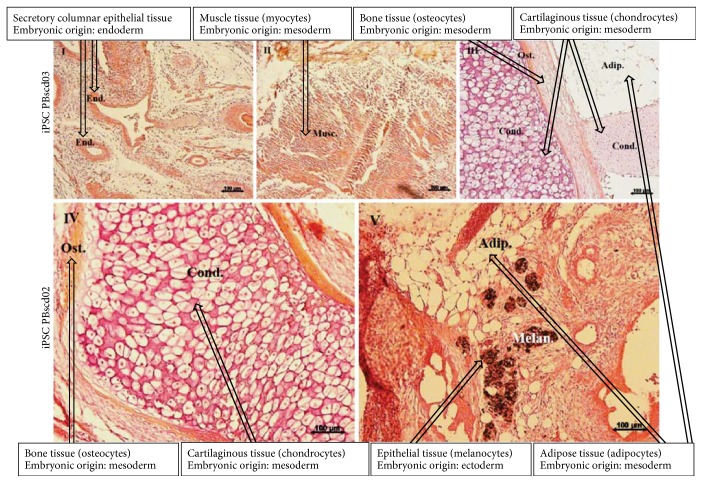
Hematoxylin and eosin (HE) staining images of histological sections of iPSC PBscd02 and iPSC PBscd03 teratomas, identifying tissue cells from the 3 germ layers. (I, II, and III) Images showing tissues originating from the mesodermal lineage, such as chondrocytes, adipocytes, osteocytes, and muscle cells, and the endoderm as columnar epithelium, with apparently secretory characteristics. (IV, V) Images identifying tissues originating from the ectodermal lineage as pigmentary epithelium consisting of melanocytes and originating from the mesodermal lineage such as chondrocytes, adipocytes, and osteocytes. Photographs obtained by light-field optical microscopy.

IPSC PBscd01 were also evaluated for in vivo differentiation ability by the teratoma formation assay. Teratomas are benign tumors formed by several cell types and tissues, considered an essential tool to evidence the efficiency of iPSC generation. Sections of the teratomas formed from the iPSC PBscd01 were processed and immunostained for cell/tissue identification of the 3 germ layers (Figure 10). We identified cells from the endodermal line, members of respiratory and glandular epithelial tissues by AFP marking (Figure 10(a), III-IV), cells of the mesodermal lineage, present in cartilage and muscle tissue by *α*-SMA marking (Figure 10(a), V-VI), and cells of the ectodermal lineage such as neural tissue (Figure 10(b), I) by positivity in marking with Nestin (Figure 10(b), II-III). The other iPSC PBscd lines were also evaluated for differentiation ability through teratoma formation assay. We also performed an immunohistochemistry (IHC) evaluation of the iPSC line PBscd08cl02^HU^ (Figure 11), as this was a clonal line, differing from the others and the iPSC PBscd01, also evaluated by IHQ. The evaluation of the iPSC PBscd08cl02^HU^ teratoma sections by IHC revealed the presence of cells/tissues of the 3 germ layers, evidenced by the *α*-SMA immunostaining positivity, identifying elongated cells like muscle fibers, of mesodermal origin (Figure 11(a), I–IV), AFP immunostaining positivity, identifying respiratory and guandular epithelial cells of endodermal origin (Figure 11(a), I and V–VII), besides the positivity for Nestin evidencing neural tissue that is of ectodermal origin (Figure 11(b)). The other lines, iPSC PBscd02 and iPSC PBscd03, were analyzed by staining with hematoxylin and eosin (Figure 12). In the iPSC line PBscd02, we observed cells derived from the endoderm, such as columnar epithelium with apparently secretive characteristics (Figure 12, I), and mesoderm, such as chondrocytes, adipocytes, osteocytes, and muscle cells (Figure 12, II-III). In the iPSC line PBscd03, we observed tissues derived from the mesoderm, such as chondrocytes, adipocytes, and osteocytes (Figure 12, IV), and the ectoderm as pigmetal epithelium composed of melanocytes (Figure 12, V).

#### 3.2.3. Prediction of the Differentiation Potential by the hPSC Scorecard Tool

The TaqMan hPSC Scorecard (Applied Biosystems) test, which contains specially formulated gene expression assays for the evaluation of iPSC and ESC, was performed as the final test for pluripotency and differentiation potential. The cells used were the undifferentiated iPSC PBscd01 and after spontaneous differentiation and formation of EB (EB iPSCscd01). For the test, RNA of both cells was extracted and quantified, and 1 *μ*g of the total RNA was used to obtain the cDNA in RT-PCR reaction. Then, the cDNA produced from both samples was used in qPCR reaction, one sample per plate, for evaluation of self-renewal potential and prediction of differentiation potential. Each plate provides a panel of pluripotency and differentiation probes, comprising the ectodermal, mesodermal, and endodermal lineage. Gene expression data were analyzed with the help of the online hPSC Scorecard analysis software.

**Figure 13:**
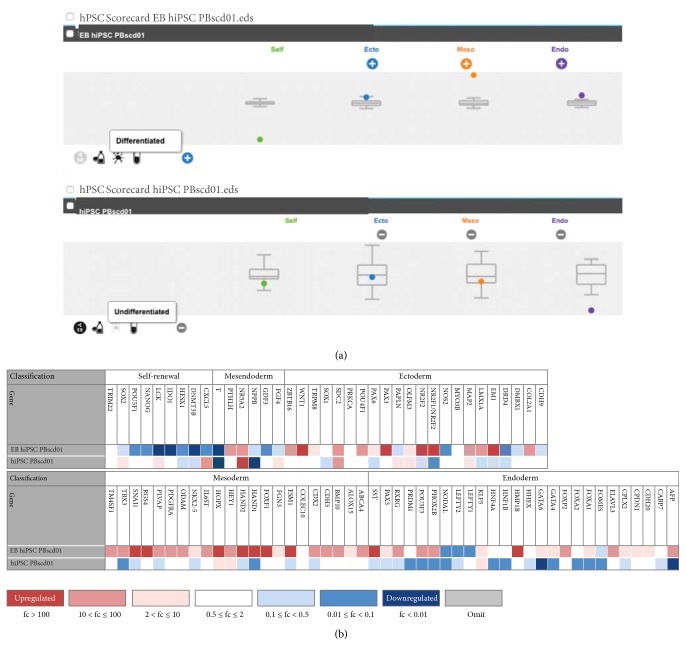
Analyses performed by the hPSC Scorecard analysis software from data of the TaqMan hPSC Scorecard test with the iPSC PBscd01 and the iPSC EB PBscd01. (a) First box: analysis of the gene expression of self-renewal and differentiation markers in EB of iPSC PBscd01, confirming its differentiated cell characteristic, with positivity for markers of the 3 germ layers, highlighting mesoderm. Second box: analysis of the gene expression of self-renewal and differentiation markers in the iPSC PBscd01, confirming its pluripotent characteristic, being negative for markers of the three germ layers, that is, differentiation markers. (b) Gene expression analysis of several markers of self-renewal and differentiation, the latter represented by gene characteristic of the 3 germ layers, ectoderm, mesoderm, and endoderm. The upper line details the EB iPSC PBscd01 experiment and demonstrates the upregulation (red, light red, and light pink) of the genes of the 3 germ layers, mainly the mesodermal, and the downregulation (dark blue, blue, and light blue) of self-renewing and pluripotency genes, in the first column. The bottom line details the iPSC PBscd01 experiment and demonstrates the downregulation of genes from the 3 germ layers, especially the endoderm, and upregulation of self-renewal and pluripotency genes.

The analysis confirmed that the iPSC PBscd01 are undifferentiated, with characteristics of self-renewal and pluripotent potential, with high expression of SOX2, and intermediate expression of NANOG and OCT4, besides high expression of CXCL5, LCK, a consensus marker of hESC, and NR5A2 which is a highly expressed gene in hESC (Figures 13(b) and 13(c), bottom line). The analysis also showed that after spontaneous differentiation, the cells give rise to cells from the 3 germ layers (Figure 13(a)), with the genes of negatively regulated self-renewal and pluripotency genes and positively regulated differentiation genes. Many highly expressed ectodermal genes, many highly expressed endoderm genes, but mainly many highly expressed mesoderm genes have been found. Of the 22 mesodermal tissue-related genes, 21 showed high expression in the EB from iPSC PBscd01 and 1 showed intermediate expression (Figure 13(c), upper line).

Together, these data demonstrate that PBscd iPSCs are morphologically, phenotypically, and functionally like pluripotent stem cells and they have potential to differentiate into different tissues from three germ layers.

### 3.3. Screening for the Occurrence of Spontaneous Integrations in iPSC PBscd

**Figure 14:**
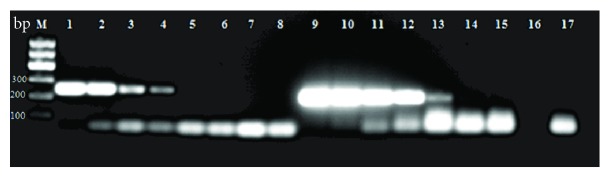
Image of the 1% agarose gel stained with ethidium bromide representative of the electrophoresis of the dilution curve of the vectors. bp, number of base pairs; M, 100 bp ladder; 1 to 7, samples of the serially diluted pEB-C5 vector from 10^7^ up to 10 times; 8, negative reaction control; 9 to 15, samples of dilutions of pEB-Tg vector, serially diluted 10^7^ to 10 times; 16, empty; 17, negative reaction control.

The iPSC PBscd were screened for the occurrence of spontaneous integrations of the vectors used for reprogramming using two sets of primers specific to each vector. Initially, we performed a test of the primers with the DNA of the pEB-C5 and pEB-Tg vectors extracted with the same kit used for the other extractions, serially diluted 10^7^ to 10 times. The dilution curve shows us how many copies of the vectors the primers can efficiently identify in the cell. A 1% agarose gel electrophoresis was performed, resulting in a vector dilution curve (Figure 14). The curve demonstrates that the primers are efficient at identifying the pEB-C5 vector up to 10^4^ copies and the pEB-Tg vector up to 10^2^ copies.

**Figure 15:**
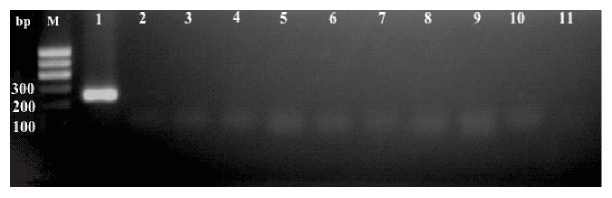
Image of 1.5% agarose gel stained with ethidium bromide representative of the electrophoresis of the pEB-C5 vector amplification reaction performed on all the generated iPSC PBscd lines. bp, number of base pairs; M, 100 bp ladder; 1, positive control MNC PBscd; 2, iPSC sample PBscd01 P5; 3, sample iPSC PBscd01 P10; 4, sample iPSC PBscd02 P6; 5, sample iPSC PBscd02 P11; 6, sample iPSC PBscd03 P4; 7, sample iPSC PBscd03 P14; 8, sample iPSC PBscd08cl02 P8; 9, sample iPSC PBscd08cl02 P9; 10, sample iPSC PBscd08cl02 P10; 11, negative reaction control.

**Figure 16:**
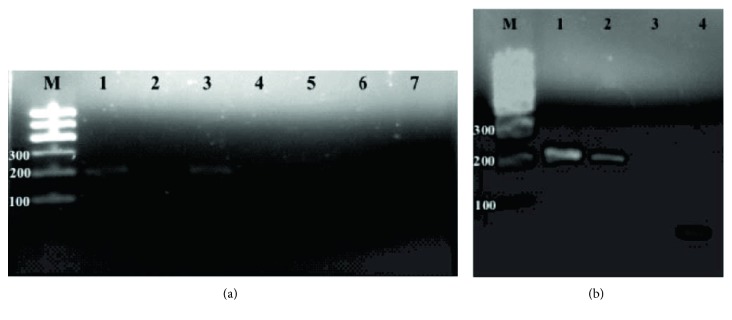
(a) Image of 1.5% agarose gel stained with ethidium bromide representative of the electrophoresis of the pEB-Tg vector amplification reaction in the iPSC lines generated PBscd. M, 100 bp ladder; 1, positive control MNC PBscd; 2, sample iPSC PBscd02 P6; 3, sample iPSC PBscd01 P5; 4, sample iPSC PBscd03 P4; 5, sample iPSC PBscd03 P8; 6, sample iPSC PBscd03 P14; 7, negative reaction control. (b) Image of 1.5% agarose gel stained with ethidium bromide representative of the electrophoresis of the pEB-Tg vector amplification reaction in the iPSC line PBscd01 to confirm vector deletion. pb, number of base pairs; M, 100 pb ladder; 1, positive control MNC PBscd; 2, iPSC sample PBscd01 P5; 3, sample iPSC PBscd01 P10; 4, negative reaction control.

For the screening of the integration of the pEB-C5 vector, a reaction was performed with at least 2 DNA samples from each line at different passages. As a positive control, the DNA extracted from the expanded and transfected parental MNCs was collected after 48 hours of nucleofection. A 1.5% agarose gel electrophoresis stained with ethidium bromide (Figure 15) was performed. The gel showed amplification of the vector only in the positive control MNC PBscd. The fragment of about 244 bp shows that the vector was still present in the cell 48 hours' postnucleofection. After the reprogramming and expansion of the cells by some passages, the vector was eliminated. For the pEB-Tg vector, a reaction was performed with the DNA samples from the iPSC PBscd lines. The same positive control described above was used. A 1.5% agarose gel electrophoresis stained with ethidium bromide (Figure 16(a)) was performed. The gel showed amplification of the pEB-Tg vector in the positive control MNC PBscd, showing that the vector was present in the cells 48 hours postnucleofection. However, amplification of the pEB-Tg vector on iPSC PBscd01 P5 (Figure 16(a), line 3) was identified. To evaluate if the vector was eliminated after cultivation for further passages, a new amplification was performed only with the DNA of the lineage of which there was positivity (iPSC PBscd01), in the passages P5 and P10. A 1.5% agarose gel electrophoresis stained with ethidium bromide (Figure 16(b)) showed amplification of the pEB-Tg vector in the positive control MNC PBscd and in the iPSC line PBscd01 P5 (Figure 16(b), line 2), as seen in the previous gel. However, the gel did not show amplification for iPSC PBscd01 P10 (Figure 16(b), line 3), showing that the vector previously found in the iPSC line PBscd01 P5 was deleted after a few culture passages.

### 3.4. Screening of Glu6Val Mutation

**Figure 17:**
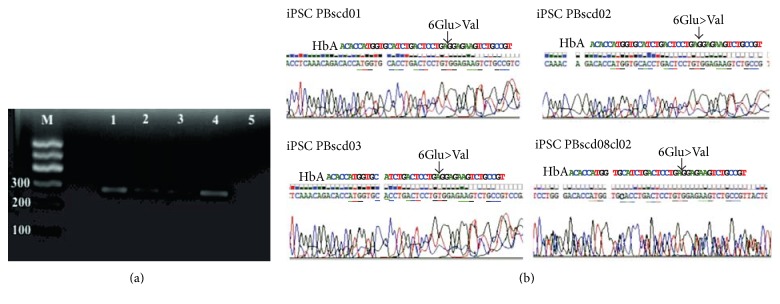
(a) Image of 1.5% agarose gel stained with ethidium bromide representative of the beta-globin gene amplification in the iPSC PBscd lineages. M, 100 bp ladder; 1, iPSC PBscd01 P5 sample; 2, iPSC PBscd02 P6 sample; 3, iPSC PBscd03 P4 sample; 4, iPSC PBscd08cl02^HU^ P10 sample. (b) Image of the sequencing data showing the mutation of the sickle cell disease, the shift of an adenine for a thymine which is responsible for the change of amino acids. The sequence of normal beta globin is arranged just above the obtained sequence, matching the nucleotides. The mutation is indicated in the figure with the arrow, and the mutated amino acid is colored in red, different from the adult normal hemoglobin, which has an adenine colored in green.

For molecular identification of the mutation responsible for the sickle cell phenotype, the generated iPSC DNA was amplified for the *β*-globin (HBB) gene with specific primers and the fragments were then sequenced to screen the mutation. A reaction was performed using a DNA sample from each generated iPSC PBscd line, and then electrophoresis was performed on 1.5% agarose gel stained with ethidium bromide (Figure 17(a)). The gel showed amplification of the HBB gene in cell lines used, without contamination or nonspecific amplifications.

Sequencing of the amplified samples in ABI 3500XL Genetic analyzer automatic sequencer was performed to locate the mutation responsible for the sickle cell phenotype. A sample of each line of iPSC PBscd generated was used. The electropherograms arranged in Figure 17(b) were obtained after analysis in ChromasPro software. The HbA sequence is shown above the sequence of the HbS mutation lines at codon 6 (GAG to GTG).

### 3.5. Hematopoietic Differentiation and Efficient Production of Hematopoietic Progenitors

**Figure 18:**
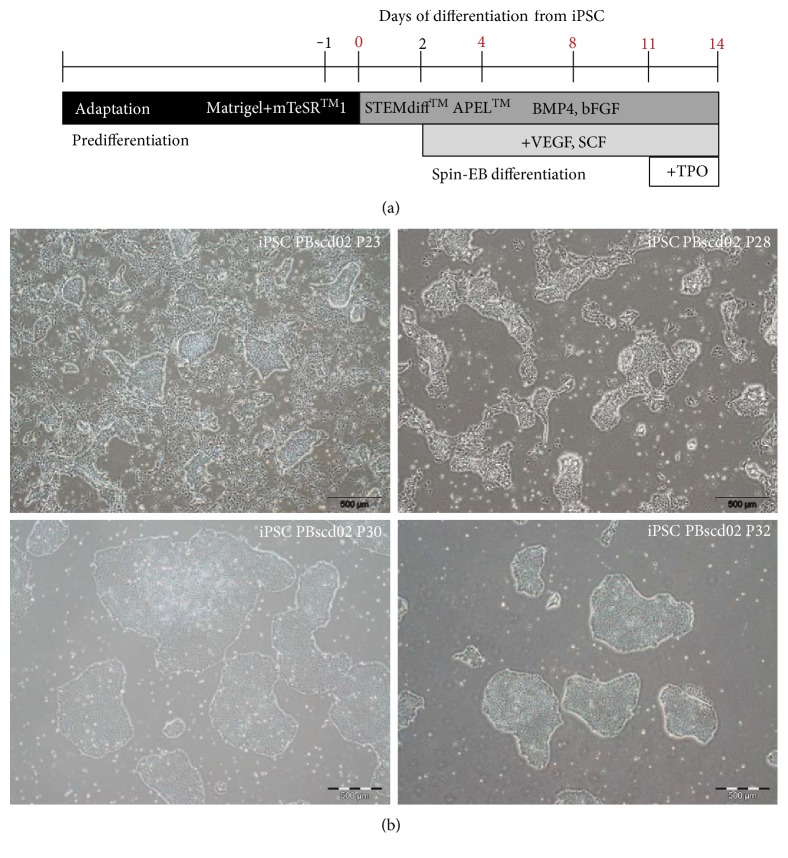
Differentiation process. (a) Schematic image representing the whole process of hematopoietic differentiation until obtaining hematopoietic progenitors, passing through a phase of adaptation of the iPSC prior to differentiation. (b) Predifferentiation process, leading the cells to adapt to the enzymatic picking. (I, II) Cells in the process of adaptation, showing some colonies, but many individualized and differentiated cells. (III, IV) Cells adapted to the enzymatic picking, showing that the colonies acquire more rounded morphology, and few or no individual cells are visible between the colonies. Images in phase contrast. Optical microscopy.

**Figure 19:**
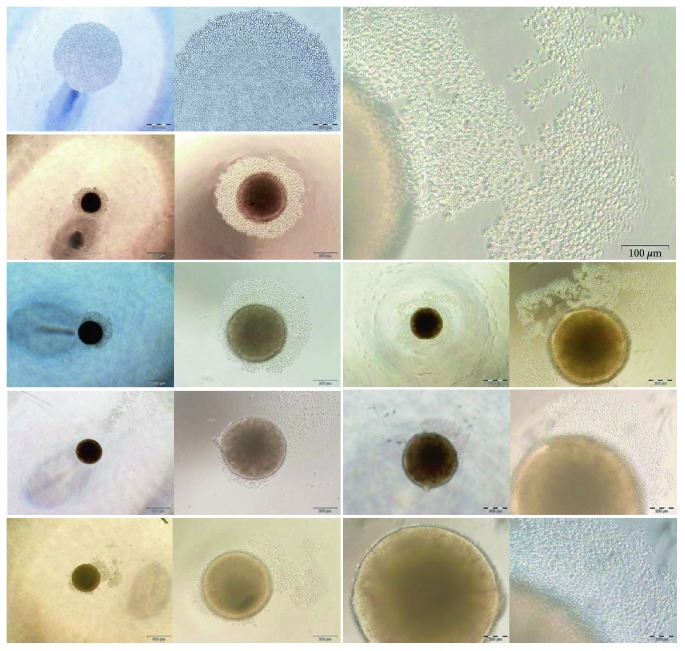
IPSC PBscd08^HU^ during the 14 days of induction of hematopoietic differentiation, showing the cell morphology, the formation of EBs, its growth, and the subsequent appearance of small cells at their edges.

Throughout this work, the hematopoietic differentiation underwent an extensive standardization to obtain an efficient protocol, which is summarized in the scheme of Figure 18(a). For this, the iPSC colonies were submitted to a process of adaptation to the enzymatic picking, so that after their individualization, the cells were regrouped in colonies and did not suffer great losses due to cell death (Figure 18(b)). The cells take at least 5 passages so that they are adapted to the enzymatic passage, but this time can vary according to the lineage employed. These adapted cells were used in the differentiation experiment to produce hematopoietic progenitors. The cells started to aggregate from the centrifugation and culture without adherence. On day D + 2, it was already possible to verify the formation of EB, with cells surrounding that did not aggregate. The monitoring of EB showed a subtle initial growth with these structures becoming noticeably more compact. On day D + 8, it was possible to verify the presence of small cells near the EB, and from day D + 11, a marked growth of the EB was observed, with the experiment being finalized on day D + 14, with the presence of small cells around the EBs (Figure 19).

**Figure 20:**
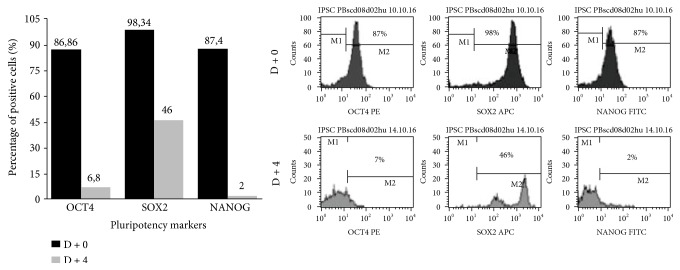
Immunophenotyping of cells obtained after induction of differentiation, by flow cytometry, showing the markers evaluated and the percentage of positive cells for each marker. On day 0, immunophenotyping of pluripotent cells prior to induction of differentiation showed the high percentage of pluripotency markers, OCT4, SOX2, and NANOG. After 4 days of induction of differentiation, immunophenotyping of the cells showed decreasing pluripotency markers. Analyses are carried out in FACSCalibur.

**Figure 21:**
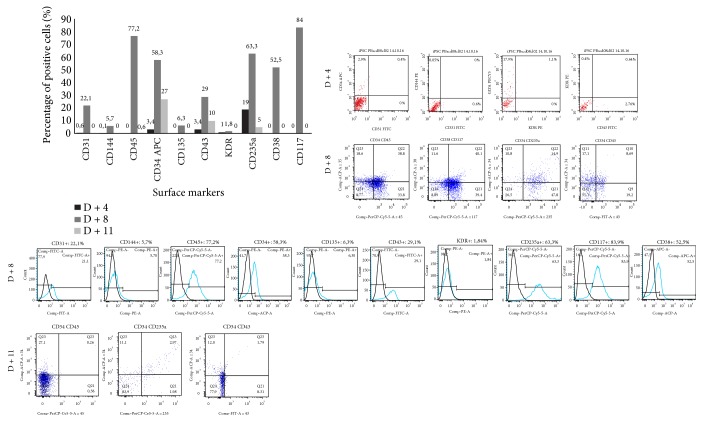
Immunophenotyping of cells obtained after 8 days of induction of differentiation by flow cytometry, showing the markers evaluated and the percentage of positive cells for each marker, as well as combinations of double positive markers. Cells were analyzed in FACSAria and analyses were performed in FlowJo software.

**Figure 22:**
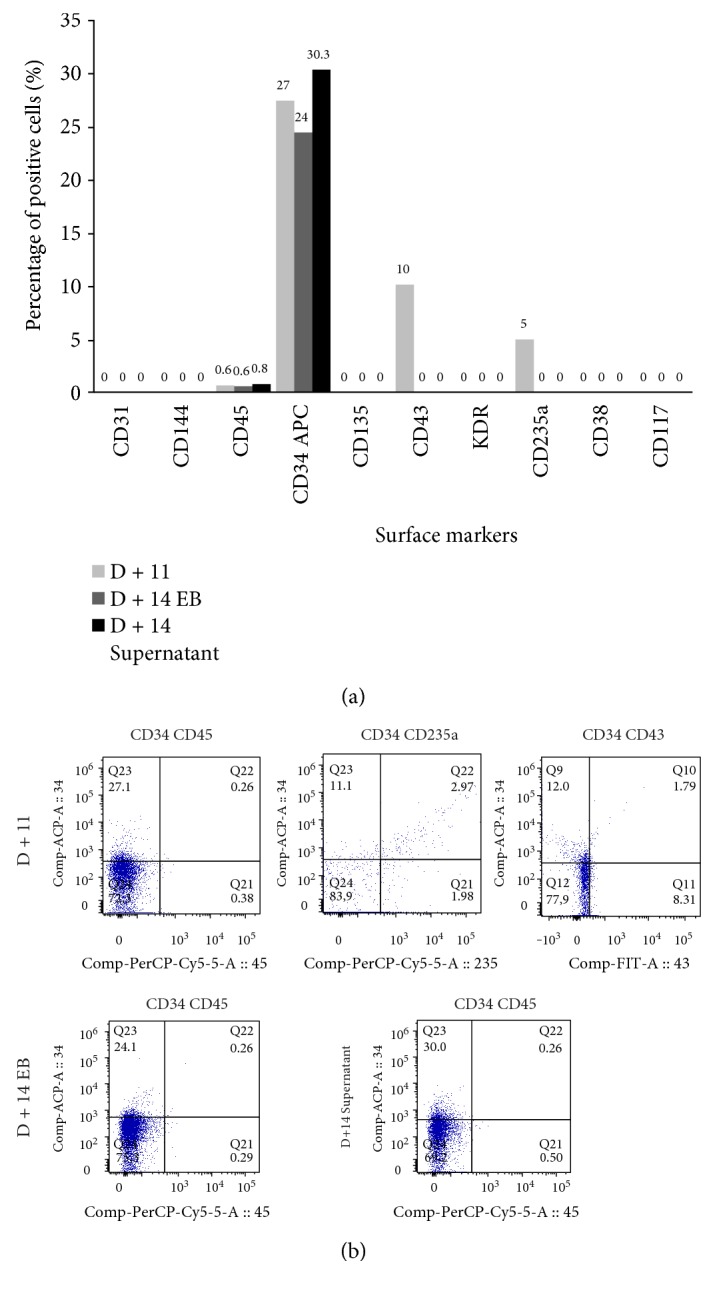
Immunophenotyping of cells obtained after induction of differentiation, by flow cytometry, showing the markers evaluated and the percentage of positive cells for each marker. (a) Immunophenotyping of the cells obtained after 11 days of induction of differentiation, showing the drop in the percentage of cells positive for markers of hematopoietic progenitors, when compared with D + 8. (b) Immunophenotyping of the cells obtained after 14 days of induction of differentiation and end of the experiment, showing loss of almost all markers, remaining only 30.3% of the CD34-positive cells when analyzed separately and 24.4% of the supernatant cells. Cells were positive for CD34 when analyzed separately. Cells were analyzed in FACSAria and analyses were performed in FlowJo software.

Prior to initiating the differentiation experiment, the differentiated cells at day 0 of differentiation (D + 0) were evaluated for pluripotency markers and showed a high percentage of cells positive for OCT4 (87%), SOX2 (98%), and NANOG (87%) (Figure 20). After 4 days of differentiation induction (D + 4), cells were evaluated for the same pluripotency markers (Figure 20) and flow cytometry evaluations showed a drop in OCT4 (7%), SOX2 (46%), and NANOG (2%) markers. After this short differentiation induction (D + 4), the EBs were also evaluated for some hematopoietic markers, such as CD31, CD144, CD34, CD43, KDR, and CD235a (Figure 21) and we noticed the emergence of still very discrete hematopoietic markers such as CD34 (3.4%) a hematopoietic progenitor cell marker, and CD43 (3.4%), a T cell and myeloid lineage cell marker. Cells were negative for KDR, a marker that shows commitment with the mesodermal lineage, but 19% of the cells were CD235a positive (glycophorin A), which is a marker of primitive erythroid differentiation. The EB collected also did not present positivity for CD31 (0.6%) and CD144 (0.05%) markers, which when expressed in conjunction with CD34, characterize endothelial cells with hemogenic endothelium potential. After further 4 days of induction (D + 8), new EBs were collected for differentiation evaluation with a panel of markers (CD31/CD144/CD45/CD34, CD43/KDR/CD235a/CD34, and CD34/CD135/CD117/CD38) (Figure 21). After 8 days (D + 8) of induction, we observed an increase in the expression of differentiation markers, with double positives as CD34^+^CD45^+^ (39%), CD38^+^CD117^+^ (40.1%), CD34^+^CD235a^+^ (15%), and CD34+ CD43+ (8.7%), in addition to high expression of hematopoietic markers such as CD45 (77%), CD34 (58%), and CD117 (84%). Cells at D + 8 were also screened for the presence of common myeloid progenitors (CMP) by the CD34^+^CD117^+^CD135^+^CD38^+^ pattern, that was not found, and the presence of megakaryocyte erythroid progenitors (MEP) by the CD34^+^CD135^−^CD117^+^CD38^+^ pattern, identified in the differentiating cells. After further 3 days of induction (D + 11), new cells were taken for differentiation evaluation, using the same panel of D + 8 (CD31/CD144/CD45/CD34, CD43/KDR/CD235a/CD34, and CD38/CD135/CD117/CD34) (Figures 21 and 22). A decrease in hematopoietic markers was observed, with some markers losing expression, such as CD31 (0%), CD45 (0.64%), and CD38 (0%); some that had low expression had the marking lost as CD144 (0%), CD135 (0%), and KDR (0%), and others had a drastic fall in the percentage of positive cells, such as CD34 (27.4%), CD43 (10%), and CD235a (4.95%). EBs also did not show double positivity for CD34/CD45 as verified on D + 8 and only 3% of the cells showed marking for CD34^+^CD235a^+^. Methocult H4434 classic cells at D + 11 were used for the CFC assay in methylcellulose (Methocult H4434 Classic) in triplicate, and after 15 days of induction, no colonies were found. On the last day of the differentiation experiment, after 14 days of induction (D + 14), some individualized cells were visible in the supernatant, which were collected along with the EB and separated for individual analysis (Figure 22). Most hematopoietic progenitor markers had been lost, with only CD34-positive cells remaining in the EB (24.4%) and in the supernatant (30.3%).

The development of the hematopoietic differentiation protocol is summarized in the Table 4, which shows the percentage of positive cells for each marker on specific days of differentiation. The table summarizes the enrichment of the population with hematopoietic progenitor cells, with a peak at day 8 and subsequent fall after that period.

## 4. Discussion

### 4.1. Reprogramming Mononuclear Cells in Integration-Free iPSC

In this study, we report that integration-free iPSC can be efficiently generated from human adult PB MNC of patients with sickle cell anemia, after brief suspension culture system, in 3-4 weeks by nucleofection of episomal vectors expressing the 5 factors OCT4, SOX2, C-MYC, KLF4, and LIN28 as a single polycistronic unit and expressing the SV40 large T antigen. These vectors are based on a well-established technology in which the inclusion of the EBNA1 gene and the OriP sequence of the Epstein-Barr virus allow a plasmid, after a single transfection, to replicate extrachromosomally, as a circular episome, in several cell types [45]. The stimulation of Tg in reprogramming was observed by other groups using different vectors and cell types [46, 47]. Nucleoporation is a method of transferring genetic material to mammalian cells considered difficult to transfect, such as stem cells and primary cells [48]. Based on the physical electroporation method, nucleoporation uses a combination of electrical parameters with specific reagents for each cell type, so that the genetic material to be transfected is transferred directly to the nucleus and cellular cytoplasm. In contrast, other commonly used nonviral transfection methods depend on cell division for the transfer of DNA to the nucleus, so nucleofection provides the possibility of transfecting even nondividing cells such as neurons and resting blood cells [49, 50]. Prior to the introduction of this technology, the transfer of efficient genetic material into primary cells was restricted to the use of viral vectors, which typically involves disadvantages as security risks due to genome insertion, complexity, and high costs.

Despite being the most used source, in the present work, the SV40 large T antigen episomal vector and the episomal vector expressing the 5 reprogramming factors in a single molecule (~18 kb) were not able to reprogram skin fibroblasts derived from patients with sickle cell anemia (data not shown). The nucleofection process is a nonviral process of nucleic acid transfer of all sizes. However, the transfection efficiency of large sequences falls considerably with increasing plasmid size, due to low cell viability [51], and this toxicity occurs only when the cells are exposed to the electrical pulses [52]. More precisely, Lesueur et al. [52] have shown that the observed drop in survival and transfection efficiency are directly linked to the physical size of each individual plasmid molecule, that is, a single copy of a large plasmid is more toxic and difficult to transfect than a single copy of a small plasmid or the number of copies of a small plasmid equivalent in mass to the single copy of the large plasmid. An option for our study would be the use of a different set of plasmids expressing the reprogramming factors, but divided into more than one molecule, to reduce their physical size, thus reducing the toxic effects to nucleofected cells.

Peripheral blood is a more advantageous source of cells than dermal fibroblasts, the most widely used source, but it requires skin biopsies, an invasive procedure that requires a long time of previous culture (more than 4 weeks). In contrast, adequate amounts of MNCs for reprogramming can be obtained from a few milliliters of peripheral blood, so that a simple venipuncture would be more acceptable to patients and donors, especially pediatric patients. The reprogramming of MNCs was simple and efficient. MNCs were cultured in specific medium with a combination of interleukins for depletion of the lymphoid population and expansion of the erythroblast/erithroid population. This stage of expansion not only drives cells into the cell cycle, so that we get a proliferative cell population, but also gives the cells certain epigenetic states that are more easily reprogrammed [42]. Chou et al. [42] have identified a DNA methylation profile of these proliferating cells more like ESC and iPSC than to nonhematopoietic cells of similar age, such as fibroblasts and endothelial cells, which could contribute to high efficiency in obtaining iPSC from these blood cells after only one transfection.

After culturing for about 12 days in a specific medium, we were able to identify the enrichment of the erythroblast/erythroid cell line population, especially regarding CD71 (transferrin receptor) levels, erythroid precursor marker [53], and CD235a (glycophorin A), an erythroid marker [54]; we also identified a decrease in the cell population expressing T cell markers such as CD4 and CD8, glycoproteins found on the surface of helper and cytotoxic T cells, respectively, as well as being found on the surface of other cells of the immune system such as monocytes, macrophages, dendritic cells, natural killer cells, among others [55]. We also identified a drop in the cell population expressing B cell markers, such as CD19 [56], and the common leukocyte antigen CD45 marker, present on the surface of most human leukocytes, such as monocytes, lymphocytes, and eosinophils, but absent in erythrocytes and platelets [57]. This variation shows that the expansion with medium and specific cytokines for lymphoid cell depletion and erythroblast/erythroid cell induction was successful in reducing the chances of reprogramming a lymphoid cell. Lymphocytes are known to lack their intact genome because of somatic rearrangements or V(D)J recombination, responsible for diversity in the repertoire of antibodies and T cell receptors [58]. IPSC obtained from nonlymphoid cells presenting their intact genome may be more suitable for therapeutic purposes, as the absence of such recombined DNA segments in the iPSC genome may eliminate some safety issues, issues raised by the observations that mice derived from reprogrammed T cells had a high incidence of T cell lymphoma [59].

The expanding cells from patients undergoing hydroxyurea treatment had an impact on cell growth, so that after consecutive falls in the first 6 days of expansion, the cells were not able to reach the initial cell population, ending on the 12th day of culture with, on average, fewer cells than at the beginning of the expansion. Hydroxyurea or hydroxycarbamide is a cytotoxic agent frequently used to treat SCA to reduce the number and frequency of pain episodes, vaso-occlusive crises, episodes of acute chest syndrome, and hospitalizations by raising fetal hemoglobin (HbF) levels [60, 61]. Its mechanism of action is the inhibition of the ribonucleotide reductase enzyme (RNR), reducing intracellular deoxynucleotide triphosphate and acting as a specific agent of phase S with inhibition of DNA synthesis and eventual cellular cytotoxicity. Its effect on HbF synthesis is attributed to the premature impairment of erythroid progenitors due to its cytotoxic suppression effects and to cell stress signaling that affects the kinetics and physiology of erythropoiesis and leads to the recruitment of erythroid progenitors with high levels of HbF [10]. Its prolonged use is associated with myelosuppression, due to its cytotoxic effects, but these same effects are beneficial for the treatment of SCA, because it reduces the production, not only of erythroid progenitors but of neutrophils and reticulocytes, which promote vaso-occlusion through adhesion to vascular endothelium, and platelets, an important mediator of inflammation [60, 62]. The effects of low cell growth found in our study can be attributed to the cytoreductive effect of hydroxyurea, which concentrates on leukocytes and, especially on erythrocytes, cells whose growth is conditioned by the cocktail of cytokines added to the culture medium.

We also identified that low cell growth found had an impact on the reprogramming of PBscd08 in iPSC, since these cells derived from patients treated with hydroxyurea did not reach the minimum number of cells ideal for the transfection of the reprogramming factors. However, hydroxyurea influenced reprogramming not only by impacting cell growth but also the number of colonies obtained; cells derived from untreated patients showed an 11-fold increase in the number of colonies obtained compared to cells from treated patients. The greatest impact on the number of colonies obtained from cells from patients treated with hydroxyurea occurred because in the cells PBscd06 and PBscd07, despite reaching the ideal number of cells for nucleoporation, no colony was obtained after culture in ESC medium. The low reprogramming efficiency of the cells of patients treated with hydroxyurea may be linked to differences in the epigenetic pattern of these cells, since hydroxyurea, as a drug that alters DNA synthesis, can lead to changes in DNA methylation pattern [63]. Although the effects of hydroxyurea are related to cell cycle inhibition leading to the activation of stress erythropoiesis, the precise mechanism by which hydroxyurea induces HbF is not fully understood. Expressions of erythroid genes of the *β*-globin and *γ*-globin loci are regulated by a complex series of epigenetic and molecular processes during development, and some epigenetic mechanisms of HbF regulation in proposed hemoglobinopathies include methylation and histone deacetylation [64]. Compared with fetal erythroid cells, the promoter region of *γ*-globin in adult erythroid cells is highly methylated, and this hypermethylation has been inversely related to HbF expression, and although hydroxyurea has not been identified as a hypomethylating agent, studies suggest a decrease in the methylation of the *γ*-globin gene in association with exposure to hydroxyurea [65, 66]. Although more experiments are needed, our results together with others cited above reinforce the need for future studies to investigate epigenetic and molecular processes as potential mechanisms of HbF expression and induction.

The iPSC PBscd lines obtained could differentiate into cells/tissues from the 3 germ layers, characteristic of pluripotent stem cells (PSCs). ESC and iPSC are defined by their potential for pluripotent differentiation and unlimited self-renewal ability [67], so that this ability to become any somatic cell type found in the human body has gained significant attention and interest in fields of cell biology and regenerative medicine [68]. In the study of these cells, quality control analyses are crucial, and pluripotency tests continue to be key components of any research project. Analyses commonly used to test such potential include quantitative PCR that search for upregulated pluripotency genes, immunocytochemistry for the detection of pluripotency markers, and formation of embryoid bodies to test the ability of differentiation into cells/tissues of the 3 germ layers in vitro [69]. However, the most effective test to evaluate the ability of PSCs to form tissues of all 3 germ layers in vivo is in the form of encapsulated tumors called teratomas [70–72]. The lines obtained from iPSC PBscd could form these structures that harbor the formation of several cell types derived from tissues with mesodermal, endodermal, or ectodermal origin, when infused in immunodeficient mice. In vivo teratoma formation is considered the most accurate pluripotency test because it provides more reliable and comprehensive confirmation than testing cells in simplified and artificial systems such as culture plates [69].

Although the teratoma formation test is considered a test of high confidence and comprehensiveness, some questions are still unanswered regarding the equivalence between ESC and iPSC, making the selection and characterization of these cells increasingly important. For example, it has been described that human iPSCs depart from ESC in the expression of hundreds of genes [73], in their overall patterns of DNA methylation [74] and in their neural differentiation properties [75]. In addition, some recent studies have argued that some iPSC lineages may retain a certain “epigenetic memory” inherited from the somatic cells from which they originated, so that they exhibit reduced differentiation efficiencies toward a specific cell type [75, 76].

To access the nature of the epigenetic variations that exist between hPSCs, Bock et al. [77] performed 3 tests with 20 established ESC lines and 12 characterized iPSC lines, and from the results, they could identify specific genes and predict the propensity of differentiation of each lineage in the 3 germinative layers. These 3 tests were combined into a bioinformatics platform that allows high-throughput predictions of the quality and utility of any iPSC lineage [77]. This platform, in scorecard form, was applied to one of our blood cell lineages, both in undifferentiated iPSC and after spontaneous differentiation in vitro. The undifferentiated iPSC analysis showed a positive regulatory pattern of CXCL5, LCK, and SOX2, a known pluripotency gene used to generate iPSC, which is at the center of the pluripotency regulatory network [78]. The lymphocyte-specific p56 protein tyrosine kinase (LCK) is considered a self-renewal marker because it was identified in the network of protein-protein interaction genes with high expression in ESC and iPSC compared to somatic cells: LCK → HM89 → CD133 → SOX2 [79]. On the other hand, CXCL5 (chemokine (CXC motif) ligand 5) is actively regulated by FGF2 (or bFGF), inducing the mobilization of hematopoietic stem cells (HSCs) from the endosteal niche or bone marrow into the peripheral blood [80, 81]. Thus, the daily addition of the cytokine *β*-FGF given by the daily exchange of the culture medium made the expression levels of CXCL5 increase considerably. Our iPSC PBscd also demonstrated positive regulation of the nuclear receptor NR5A2, previously related to the reprogramming of murine somatic cells in iPSC, replacing OCT4 [82]. Heng et al. [82] demonstrated that NR5A2 shares a lot of common gene targets with SOX2 and KLF4, suggesting that all three transcription factors work in combination with reprogramming.

The analyses of EB formed by the spontaneous differentiation of one of the iPSC PBscd lines demonstrated a shutdown of genes related to self-renewal and an upregulation of genes related to the 3 germ layers, mesoderm, endoderm, and ectoderm. However, we could observe that the genes related to the mesoderm were more expressed and 95.5% were upregulated, which did not occur with the other groups (Ecto: 64%, Endo: 61.5%). Retention of a donor cell “memory” in iPSCs seems to make it easier to redifferentiate them in the donor cell type than in another cell type [83–85]. Kim et al. [83] demonstrated that iPSCs derived from both blood and dermal cells have distinct potentials for both hemopoietic and osteogenic direction, respectively. IPSCs derived from blood cells form hemopoietic colonies more easily, while iPSCs from dermal cells form more colonies when differentiating in the osteogenic direction [83]. However, some studies have demonstrated that a unique pattern of methylation and/or gene expression characteristic for low-pass iPSC is not stable but gradually reverts to the pattern of ESC gene expression as consequence of culture passages [85, 86]. In the case of iPSC PBscd, the finding that identifies a possible “memory” of the blood cells from which the iPSCs were derived could facilitate hematopoietic differentiation, which would be an advantage, considering how complex this differentiation may be. One of the overexpressed genes in EB from iPSC PBscd that stood out from the mesodermal lineage was SNAI2, also known as SLUG, a zinc finger transcriptional repressor that downregulates E-cadherin expression in neural crest premigratory cells, from which induces epithelial cells to lose the strong binding characteristic to acquire a mesenchymal phenotype [epithelial-mesenchymal transition (EMT)], allowing gastrulation in the embryonic development [87]. The transition from a mesenchymal state to an epithelial state (MET) is known as a mandatory phase during the early stages of reprogramming [88, 89], as well as the reverse path, EMT is essential for efficient differentiation, whose occurrence in EB reflects the EMT observed during gastrulation in the human development [90].

In summary, the Scorecard methodology allowed us a rapid lineage-specific characterization and allowed us to identify how well the iPSC PBscd can differentiate into mesodermal lineages such as hematopoietic tissue and in the 3 germ layers. All the information provided by the test requires a more thorough evaluation, and all derived lineages in the same way must be compared to each other to identify lineage-specific variations.

Using PCR (2 sets of specific primers), we did not detect the vector DNA either as episomes or anywhere in the iPSC genome analyzed, after about 10 passages. Similar results were found by Chou et al. [42], where plasmid DNA became undetectable after 10–12 passages. The use of the plasmidial system for reprogramming as well as being a nonintegrative system also provides some advantages over other nonintegrative or nonviral methods, such as ease of preparation and stability, different from proteins and mRNA, which require repetitive transfer of multiple proteins and mRNA daily for up to 17 days [91, 92].

### 4.2. Efficient Differentiation of iPSC for Generation of Hematopoietic Progenitors

We have developed a simple and efficient method of obtaining hematopoietic progenitor cells from the combination of a methodology of forced cell aggregation and formation of EB with induction through the addition of specific cytokines. Davis et al. [93] demonstrated the role of BMP4 in inducing mesoderm from ESC, which is also dependent on Activin A and bFGF signaling [94, 95], and several studies have shown the role of BMP4, SCF, bFGF, and VEGF in promoting hematopoiesis from ESC [96–99].

Cells after induction did not show KDR marking and showed positivity to 31 only after 8 days of induction. However, CD31, which was initially identified in endothelial cells and platelets [100], is also present in leukocytes [101], which could also explain the presence of this marker in differentiating cells. The hemogenic endothelial phenotype was defined as KDR^+^CD117^+^CD45^−^ cells [102–104] and demonstrated that these cells give rise to multilinear hematopoietic progenitors which are KDR^−^CD117^+^CD45^+^ cells and can be distinguished from mature blood cell types having the KDR^−^CD31^±^CD45^+^. On day 8 of differentiation induction, we observed KDR^−^CD45^+^ populations, both CD117^+^ and CD31^±^; however, before day 8, we did not observe KDR^+^ cells. The KDR receptor appears in cells at an early stage of mesodermal differentiation [105] and may not have been detected because we did not evaluate every day of differentiation; after the initiation of the differentiation, we only performed the evaluation after 4 days of induction, and after another 4 days, and given the rapid production characteristic of progenitor cells (only 8 days), this marking may have been lost.

On day 8 of differentiation induction, we observed that the cells presented as a CD34^+^CD45^+^ and CD34^+^CD43^+^ population. The CD34 marker can be expressed in a wide variety of cell types, so that their use alone may not be sufficient to identify a pure population of hematopoietic progenitors from hPSC [106]. Our population demonstrated CD43 positivity, a marker expressed in the T cell cytoplasm and in myeloid lineage cells, whose marking clearly separates CD43^+^ hematopoietic colony-forming progenitors from CD43^−^CD31^+^ endothelial cells and CD43^−^CD31^−^ cells with mesenchymal characteristics [107, 108]. In cultures of hPSC differentiated with OP9, the first CD43^+^ cells were detected within VE-cadherin^+^ cells near day 4 of differentiation [107–109]. These cells, CD41a^−^ and expressing the erythroid marker CD235a (glycophorin A) and low levels of CD43, were defined as angiogenic hematopoietic progenitors, which maintained the ability to produce endothelial cells [109, 110]. On day 4 of differentiation, our cells expressed CD235a (glycophorin A) and low CD43 levels; however, we did not evaluate the CD41a marker on day 4. On day 11, we performed the methylcellulose assay, but the cell had already lost its progenitor cell potential, leaving only the CD34 marking at higher levels.

We could not observe many morphological changes happening during the differentiation process because this process occurred in the EB. However, different from monolayer differentiation protocols using stromal cells such as OP9 cells, EBs can mimic a microenvironment more favorable to development and differentiation. Differentiation from EB can be considered more like what happens in the hematopoietic niche, supporting the development of hematopoietic progenitor cells, since the process of hematopoietic differentiation requires intrinsic and extrinsic signaling and hierarchical organization of hematopoietic precursors [111].

In summary, the results of the differentiation show that the cells, obtained in greater quantity on day 8 of differentiation, are hematopoietic progenitor cells coming from a hemogenic endothelium. However, further analysis must be performed to prove this pathway of induction. Thus, our data show that iPSCs are also capable of differentiating into hematopoietic progenitor cells and our method of differentiation has shown promise.

## 5. Conclusion

This work demonstrated the establishment of iPSC lineages derived from patient cells with sickle cell anemia, that is, a cell with a genetic background that is able to self-renew indefinitely and can differentiate in vitro in any cell type. Thus, we provide a valuable tool for a better understanding of how the disease occurs and the possible causes for its clinical discrepancies among the patients affected, in addition to making possible the development of new drugs and more effective treatments for the disease and to provide a better understanding of the widely used treatments available, such as hydroxyurea. The development of an efficient protocol to obtain hematopoietic progenitors, besides contributing to the concept of modeling the disease, allows us a better understanding of the process of hematopoietic differentiation, a complex and not yet fully clarified system. The great difficulty in establishing robust protocols for hematopoietic differentiation is mainly due to the lack of understanding of this process that occurs in the microenvironment of the bone marrow and the difficulty to artificially mimic this complex microenvironment in the culture plate.

## Figures and Tables

**Table 1:** Set of primers used in the screening of spontaneous integration of the vectors used to reprogram iPSCs.

Primer	Sequence	Vector	*μ*g DNA	Ta (°C)
EBNA_1 forward	5′-TTTAATACGATTGAGGGCGTCT-3′	pEB-C5	0,1	57
EBNA_1 reverse	5′-GGTTTTGAAGGATGCGATTAAG-3′	pEB-C5		
Tg foward	5′-GCCAGGTGGGTTAAAGGAGC-3′	pEB-Tg	0,02	60
Tg reverse	5′-GGTACTTATAGTGGCTGGGCTGT-3′	pEB-Tg		

Ta: annealing temperature.

**Table 2:** Efficiency of reprogramming of MNCs by episomal vectors, showing the details of the 6 experiments.

iPSC line	Cell source	Treatment with HU	Age	Number of transfected cells	Days of expansion	Number of colonies	First picking	Reprogramming efficiency (%)
PBscd01	HbSS (F)	No	20	2,5 × 10^6^	14	24	Day 14	0.0075
PBscd02	HbSS (F)	No	28	2,5 × 10^6^	14	12	Day 15	0.0046
PBscd03	HbSS (M)	No	30	2,0 × 10^6^	12	5	Day 15	0.0042
PBscd06^HU^	HbSS (M)	Yes	32	2,5 × 10^6^	14	No colonies	—	0
PBscd07^HU^	HbSS (M)	Yes	28	2,4 × 10^6^	14	No colonies	—	0
PBscd08cl02^HU^	HbSS (M)	Yes	19	1,9 × 10^6^	14	4	Day 18	0.0011
PBscd08cl03^HU^	HbSS (M)	Yes	19	1,9 × 10^6^	14	4	Day 18	0.0011
PBscd08cl04^HU^	HbSS (M)	Yes	19	1,9 × 10^6^	14	4	Day 18	0.0011

PB: peripheral blood; scd: sickle cell disease; Hb: hemoglobin; S: mutated hemoglobin; F: female; M: male; HU: hydroxyurea.

**Table 3:** Summary of characterization carried out for each iPSC line.

iPSC line	Cytometry analysis	qPCR pluripotency	Pluripotency immunocytochemistry	EB formation/qPCR	Teratoma formation	IHQ analysis	HE analysis	Scorecard
PBscd01	OCT4/NANOG/SOX2	OCT4/NANOG/SOX2	OCT4/NANOG/SOX2	NESTIN/*α*-SMA/*α*-FETO	FORMED	ECTO/MESO/ENDO	STAINED	CARRIED OUT
PBscd02	OCT4/NANOG/SOX2	NO	NO	NO	FORMED	NO	STAINED	NO
PBscd03	OCT4/NANOG/SOX2	NO	NO	EB FORMATION	FORMED	NO	STAINED	NO
PBscd08cl02^HU^	OCT4/NANOG/SOX2/SSEA4	NO	NO	EB FORMATION	FORMED	ECTO/MESO/ENDO	STAINED	NO
PBscd08cl03^HU^	OCT4/NANOG/SOX2/SSEA4	NO	NO	NO	NO	NO	NO	NO
PBscd08cl04^HU^	OCT4/NANOG/SOX2/SSEA4	NO	NO	NO	INJECTED	NO	NO	NO

PB: peripheral blood; scd: sickle cell disease; HU: hydroxyurea; EB: embryoid body; *α*-SMA: alpha-smooth muscle actin; *α*-FETO: alpha-fetoprotein; IHQ: immunohistochemistry; ECTO: ectoderm; MESO: mesoderm; ENDO: endoderm; HE: hematoxylin and eosin.

**Table 4:** Summary of percentages of positive cells for each marker on specific days of differentiation.

Markers	D + 0	D + 4	D + 8	D + 11	D + 14 EB	D + 14 supernatant
OCT4	86.86%	6.8%	—	—	—	—
SOX2	98.34%	46%	—	—	—	—
NANOG	87.4%	2%	—	—	—	—
CD31	—	0.6%	22.1%	0%	—	—
CD144	—	0.1%	5.7%	0%	—	—
CD45	—	0%	77.2%	0.6%	0.6%	0.8%
CD34 APC	—	3.4%	58.3%	27%	24%	30.3%
CD135	—	0%	6.3%	0%	0%	0%
CD43	—	3.4%	29%	10%	0%	0%
KDR	—	1%	1.8%	0%	—	—
CD235a	—	19%	63.3%	5%	0%	0%
CD38	—	—	52.5%	0%	0%	0%
CD117	——	—	84%	0%	0%	0%
CD41a	—	—	—	—	0%	0%
CD42a	—	—	—	—	0%	0%

EB: embryoid body; APC: allophycocyanin; KDR: kinase insert domain receptor.
